# Understanding and Fostering Mental Health and Well-Being among University Faculty: A Narrative Review

**DOI:** 10.3390/jcm12134425

**Published:** 2023-06-30

**Authors:** Dalal Hammoudi Halat, Abderrezzaq Soltani, Roua Dalli, Lama Alsarraj, Ahmed Malki

**Affiliations:** Academic Quality Department, QU Health, Qatar University, Doha P.O. Box 2713, Qatar; asoltani@qu.edu.qa (A.S.); rdalli@qu.edu.qa (R.D.); lama.alsarraj@qu.edu.qa (L.A.); ahmed.malki@qu.edu.qa (A.M.)

**Keywords:** mental health, stress, burnout, anxiety, depression, faculty, well-being

## Abstract

In recent years, there has been increasing recognition of mental health concerns in academia, with stress, burnout, anxiety, and depression being reported among faculty members. The demanding work environment, the need to balance personal and professional duties, and the constant pressure of productivity while navigating multiple tasks of teaching, research, mentorship, professional development, and service all impact the mental health and overall well-being of faculty. Higher education institutions have structurally changed as has the research landscape. These changes as well as faculty-specific and student-specific factors coupled to the effect of the COVID-19 pandemic have led to profound effects on the mental health of academics. This paper is a narrative review of the pertinent literature describing faculty mental health and well-being. It summarizes the available evidence on factors influencing faculty mental health and shows the prevalence of anxiety, depression, stress, and burnout among faculty from various academic fields and along the whole academic ladder. Using a suggested framework that collates the efforts of leaders and faculty, the paper concludes by exploring strategies that promote work–life balance among academics and suggesting effective interventions to improve their mental health outcomes.

## 1. Introduction and Overview of Mental Health Issues

Across academia, faculty members remain the principle assets of the educational process, where they influence individuals, groups, and the society and cater to the demands of producing capable and efficient human resources that meet the needs for community and employment [1]. A dedicated and accomplished group of individuals shaping young minds, conducting groundbreaking research, and advancing knowledge [2], faculty may face a hidden struggle with demanding workloads, intense expectations, teaching excellence, and administrative duties. The pressure to secure their career path and navigate a highly competitive academic landscape can indeed impact faculty members’ mental well-being [3]. As such, the mental health of academics is a pressing concern that demands attention, and this may exhibit repercussions on individuals and institutions [4].

Over the past decade, there has been substantial interest in mental health issues, with mental health conditions being increasingly recognized among the leading causes of disease burden [5,6]. The World Health Organization (WHO) considers mental health a basic human right and defines it as a state of mental well-being that enables individuals to cope with the stresses of life, realize their abilities, learn and work efficiently, and contribute to their community and to socio-economic development [7]. In the definitions of the Centers for Disease Control and Prevention (CDC) [8] as well as the National Institutes of Health (NIH) [9], mental health includes persons’ emotional, psychological, and social well-being and affects how they think, feel, and act. It also plays an essential role in determining how individuals handle stress, relate to others, and make healthy choices. Mental health is crucial at every stage of life from childhood to adulthood, with a vital importance equal to that of physical health [10].

In 2022, the WHO released its largest-ever review on mental health since the turn of the century, describing mental health as “a growing crisis”. The report highlighted that approximately one billion individuals, i.e., 12.5% of the world population, live with a mental disorder, that suicide accounts for over 1% of deaths, and that mental health conditions cause 1 in 6 years lived with disability. As such, the report called for an urgent transformation to address mental health, stressing that maintaining the status quo is simply ineffective [11]. Moreover, the rapid global spread of COVID-19 pandemic and its tremendous impact on millions of lives, the economy, healthcare, education, and the environment [12] is likely to have impacted people’s mental health profoundly. Within the first year of the pandemic, there was a 25% increase in the prevalence of depression and anxiety worldwide [13], with documented worsening of psychiatric symptoms [14], especially during lockdown, with the associated social isolation [15,16]. About 10% of the global population experienced ongoing distress in the aftermath of the pandemic [17], with women, young individuals, those with pre-existing mental or physical issues, and minority groups being the most vulnerable [18].

The burden of mental health is multifaceted. First, social stigma associated with mental health conditions leads to concealment and reluctance to seek professional specialist support [19,20]. This becomes even more prominent when other social determinants play a role, such as lower socioeconomic classes, individuals with barriers to mental health services, those in economic crisis conditions, and those who lack social support from family and friends, who generally have lower indicators of good mental health [21,22,23]. Among the direct consequences of this are the increased risk of transitioning to psychosis in addition to poor clinical outcomes [24]. Mental health conditions also indirectly impact employment, education, health, social care, and welfare benefits [25]. Second, the implications of mental health conditions on health and disease remain substantial. In a systematic analysis of global disease burden published in 2022, mental health conditions remained among the top ten leading causes of disease burden worldwide, with no evidence of global reduction since 1990. The proportion of global disability-adjusted life-years (DALYS) due to mental health conditions increased from about 3% to almost 5% in about 20 years [6]. Third, the economic encumbrance from mental health conditions is particularly concerning. Decrease in productivity due to mental health conditions costs the global economy USD 1 trillion annually, and poor mental health with reduced productivity were estimated in 2010 to cost the world economy approximately USD 2.5 trillion per year, a sum projected to rise to USD 6 trillion by 2030 [26]. Nevertheless, mental health expenditure accounts for less than 2% of government health budgets on average [27]. Given these current and anticipated impacts of mental health, there is a need to develop better understanding of the trajectory and burden of mental illness to allocate resources for more effective interventions [28].

The surge in mental health issues and needs has indeed spread to academia, with reports of increasing stressors and emotional effects experienced by university faculty [29]. Despite the numerous benefits associated with a career in academia, it often involves responsibilities that can influence well-being and increase susceptibility to psychological distress and poor mental health [30]. This narrative review aims to examine the existing evidence regarding work-related psychosocial stressors among faculty and to highlight factors contributing to their mental health concerns. In this research, the narrative review process was based upon searches conducted on *PubMed* and *Google Scholar* for the relevant literature examining the mental health of faculty. Keywords including faculty, academics, mental health, well-being, depression, anxiety, stress, and burnout were used. Studies from different universities addressing the mental health of faculty members were included, while studies not addressing mental health or studies in different populations were excluded. The extracted data were then organized and summarized to present key issues in faculty mental health. The review also presents frameworks to improve mental health among academics, building on the existing knowledge to determine future research directions.

## 2. Mental Health in Academia

### 2.1. Rising Concerns about Mental Health among University Populations

A growing body of research has recently been accumulated to document mental health concerns among university populations, which has become a priority for academic institutions [31,32]. Recent evidence from 2023 indicates that one-third of the campus community, including faculty, staff, and students, experiences symptoms consistent with depression, anxiety, and/or stress [33]. Currently, the higher education community has a better understanding of how mental health issues impact academics given the competitive environment. As such, unraveling and addressing mental health in academia is vital for the well-being of current and future scientific leaders and researchers [34].

Among undergraduate students, studies have demonstrated 10–58% prevalence rates of depression, 15–52% prevalence rates of anxiety, and up to 40% prevalence of suicide-related outcomes (such as suicidal ideation, attempts, and behaviors) [35,36,37,38]. Graduate students experience similar issues, with their journey through academia being inundated with obstacles and difficulties that often impact their mental health and result in stark consequences on well-being and personal, academic, and career success [39]. For instance, a survey of Ph.D. students reported that over 75% work more than 40 h per week, and nearly 40% are dissatisfied with their work–life balance [40]. With the COVID-19 pandemic and associated disruptions, the issues of poor mental health among university students further ascended, according to the findings of a survey of over 30,000 undergraduate and 15,000 graduate students at nine universities in the U.S. [41], with almost double the rates of anxiety and depression. Furthermore, Wang and Colleagues [42] reported that stress and anxiety levels among at least 70% of university students rose during lockdown, and less than half were able to adequately cope with stress brought about by the pandemic. The numerous educational challenges associated with COVID-19, such as communication issues, increased isolation, limited access to support services, disruption of curricula, as well as inequalities in virtual learning environments, all had their toll on students, with negative effects on mental health [43]. These and other findings [44,45,46,47,48] necessitate better preventive measures for mental health in higher education and require mitigation strategies that produce a persistent impact on university students’ mental health and well-being as a component of preparedness for future outbreaks. Such strategies as mental health programs, well-being initiatives, and student counseling shall become more crucial in light of recent findings that link the mental health of university students to academic achievement, success, and motivation. Research in this regard shows that students with more a balanced mental health status have higher levels of motivation to study, and those with lower academic performance have less favorable mental health [31,49,50].

Apart from students, technical-administrative staff at universities have their own mental health challenges. In a study from Brazil, Borges and colleagues [51] showed that the mental health of non-teaching technical and administrative staff was affected by contract and legal conditions, and common psychological alterations were identified. Furthermore, another study revealed that instability of working conditions, work overload, lack of employees, and absence of performance evaluation were among the frustrations those workers experienced in their daily routine [52]. As such, the seriousness and complexity of mental health issues may affect students, staff, and faculty (discussed shortly) across universities and are connected with physical health, sleep patterns, and nutrition [53]. Suitable initiatives, services, and resources should ensure that work-environment stress is alleviated for all these parties. Although some initiatives are ubiquitous and can enhance the well-being of different universities and populations, it should be kept in mind that every campus is unique, and its particular experiences should be specifically considered to address its mental health needs.

### 2.2. Focus on University Faculty and Data Regarding Their Mental Health

Although there is plenty of literature on university students’ mental health, there is paucity of data examining the mental health of faculty despite the importance of this topic. This may be attributed to several factors such as stigma, lack of awareness, limited research focus, and privacy [33]. In 2020, an associate professor and researcher from the École Polytechnique Fédérale de Lausanne (EPFL), after two heart attacks in three years, published a feature article addressing the mental health of faculty [54]. He described the overwhelming situation of young scientists moving on to assume faculty positions with different tasks of teaching, mentoring, leadership, publication, funding, deadlines, administrative duties, and service. All of these within a hypercompetitive, perfectionist culture result in a status quo described as a “storm of academia”, with faculty having a “too high price to pay”, and because of this, in reality, they are experiencing frequent stress, burnout, and insecurity, as are their students.

In parallel to this feature article, reports on the mental health of faculty have eventually risen in numbers to provide further insights into a topic that is not clearly defined in the literature [55]. For example, in a systematic review on research professors [56], 20% experienced stressful psychological variables such as lack of control and emotional fatigue caused by overload, multi-tasking, demanding evaluation systems, complexities of the tenure track, and lack of recognition. Smith and colleagues [55], in their qualitative, in-depth analysis on barriers to seek help for mental well-being among faculty, indicated that academic culture, influence of leadership, personal mental health experience, interpersonal dynamics, stigma, and competitiveness can all prevent faculty from seeking support. These and other findings [57,58,59,60] drew attention to the unusual and growing stressors that faculty encounter due to excessive workload, lack of control, limited autonomy, insufficient resources, poor support, multiple conflicting work and personal responsibilities, and work–life imbalance [61,62]. Some of the many symptoms that the faculty report include depression, anxiety, suicidal ideation, unfulfillment, frustration, isolation, difficulty concentrating, lack of motivation, irritability, and sadness. In addition, some somatic signs such as headache, gastro-intestinal upset, hypertension, and heart attacks were precipitated by the cultures and working circumstances of academia or severely aggravated by them [29,54,63]. Despite high levels of mental discomfort across higher education, university staff often feel unable to disclose mental health issues, indicating a lack of familiarity with support structures as well as fear of stigma and discrimination [64]. Accordingly, faculty avoid seeking help, as they would give the impression of weakness and inadequacy, a perception that may be detrimental to their career [65].

In summary, there are existing observations of profound and widespread mental health concerns that cannot be overlooked, are affecting academics, and are sometimes described as a crisis [29,34,66]. Accordingly, there is a need to more entirely recognize, analyze, and moderate the occupational stressors that adversely affect the academic workforce through a comprehensive, career-wide research approach. Such an approach is necessary to build foundations for the future viability of an educational profession dependent on strong and resilient academics [67]. This becomes especially imperative when higher education institutions understand that their students’ success, mental health, and well-being are robustly contingent on those of their educators, advisors, and mentors [63]. In the following sections, this review describes evidence-based predisposing factors to mental health issues among faculty and available reports on the prevalence and consequences. It also highlights practices and solutions that may be attempted to positively impact the well-being of faculty and their mental health attributes in the workplace.

## 3. Review of Factors Contributing to Mental Health Concerns among Academics

Mental health is never simply an individual concern but rather largely influenced by many personal, cultural, and social elements that interact for reshaping the concepts, discourse, and practices of well-being [68]. This applies to faculty mental health, which could be affected by factors related to the faculty themselves, to their students, or to particular environmental (such as the COVID-19 pandemic) or organizational (i.e., university) changes.

### 3.1. Faculty-Related Factors

A career in academia can offer a long-lasting, inspiring, and rewarding experience. It is also an opportunity for professional growth, teaching, generating knowledge, and mounting personal accomplishments. All these gains, however, never occur without challenges [69]. It is sometimes a common misperception that faculty members are merely teachers [70]. Despite this general belief, faculty at most higher education institutions are expected to teach students, collaborate with peers, realize research, and serve their university and their community. With such a wide range of duty expectations and multiple tasks, an intriguing dynamic state is produced where mental health and well-being may be affected [60].

For early-career faculty, the stressful preliminary years typically occur at the same time a faculty member is building a personal life, starting a family, or caring for young children or for aging or ill parents [71]. At such early stages in their job path, and while trying to develop their career identity [72], junior faculty struggle with contradictions between traditionally rewarded behaviors and those required to achieve greater diversity, relevance, and engagement in the academy. They face the issue of projecting an idealized image of academics and living “the life of the mind”, precluding them from taking care of their hearts and bodies and resulting in compromised health, anxiety, depression, and family issues imposed by job stress [73]. Recently, work–life balance as studied among early-career faculty was consistently lower than that reported by faculty at higher ranks [74]. Likewise, Azevedo and colleagues [75] showed that stress has a significant and direct impact on the work–life balance satisfaction of junior faculty and encouraged universities to attend to this young group in order to maintain the reputation of their programs as well as the faculty scholarly achievements, educational contributions, and service undertakings. Additionally, for early-career researchers, such as those in post-doctorate positions, mental health is affected by lack of funding, mobility needed to diversify career trajectories, productivity pressure, and lack of job security [76]. This population is also significantly affected by research burnout, a feeling of mental tension and emotional exhaustion mounting from the pressure to accomplish demanding research tasks in a limited time. A they are concerned about enhancing their professional reputation, leaving a permanent fingerprint in their field, and improving their financial status, these faculty end up suffering heightened stress levels, devote less focus to teaching, and may produce research that lacks relevance and innovation [77]. In 2020, *Nature* published the first ever survey on postdocs, showing that 32% reported their experience to be worse than they expected, 56% had a negative view of their career, and fewer than half would recommend a scientific career to their younger self, expressing unease and insecurity among this group of early researchers [78].

As faculty advance through their academic careers, they develop through the mid-career phase mostly as associate faculty, who comprise the largest segment of the academic profession [79]. At this level, the faculty set new goals, switch roles, consolidate their careers, and concentrate on productivity and creativity in areas meaningful to their academic path. They profit from programs that train new capacities such as leadership skills, which are beneficial to them [72]. Mid-career faculty also create and maintain niches of community engagement and commitment to societal service and change [80]. The literature suggests that supporting mid-career faculty through a facilitative and flexible job environment is key to university success [81]. Yet, this population tends to be the least satisfied among academics, with layers of high expectations and increased demands. Many mid-level faculty report a professional crisis or apex during their mid-career, resulting in the questioning of personal and professional identity. They also find less frequent opportunities for mentoring, feedback, and professional development and express feelings of burnout and isolation [82,83]. Moreover, the lack of clarity regarding what is expected from faculty members and the requirements for career progression and promotion additionally aggravate the situation [63]. Reports exist of low psychological well-being among this group due to high job demands [84], challenges in dealing with an increasing generational gap between oneself and one’s students, and feelings of fatigue [85]. Moreover, mid-career researchers suffer some ongoing fear that employment is dependent on the outcome of funding or securing grants, placing them under extensive pressure, which ultimately impacts their well-being [86,87].

At the professorial level, senior faculty reach the top of their academic hierarchy, rendering them free from promotional pressure and the associated toll it brings [29]. These academics can significantly impact institutional vitality, and their disengagement can impair an entire academic unit [88]. The professional needs of senior faculty often differ from those of their early- or mid-career colleagues. Although some may still be interested in pursuing career goals, they become more focused on new aspects of their profession, such as contribution to teaching, administrative roles, service, mentoring of junior faculty, as well as progressing their research agendas. Many are also interested in more flexible work schedules and in serving the academy in areas where they may have well-developed aptitudes and expertise [89]. In a survey of senior family-medicine faculty, 55% considered pursuing other options or a career transition, 67% planned to retire by age 66, and 43% reported some degree of career dissatisfaction, which was significantly more likely in males employed in academic settings [90]. Furthermore, 67% of academic-medicine senior faculty reported frequent issues with balancing personal and work time, 66% had concerns of maintaining health, 51% were not receiving mentoring of any kind, and 47% reported they would like to have a mentor [91]. As such, senior faculty have special career concerns and needs; given that these faculty can be valuable resources even after retirement, specific programs to meet their needs and maintain their mental health and well-being are mandatory.

Therefore, it is evident that at the various phases of the academic track, the lives of faculty are always overwhelmed with an abundance of personal and professional responsibilities, although the nature of these responsibilities may differ. To remain passionate educators and scientists, to feel ultimate satisfaction from having completed their work, and to receive recognition from peers and institutions, faculty struggle with maintaining good mental health and balancing life with work. Given that faculty members’ busy and stressful lifestyles impede their ability to uphold their various responsibilities, the mental health crisis in this population should be acknowledged and meticulously addressed. Moreover, considering the repercussions of faculty mental health issues and the ripple effect these may exert on the entire academic community, including students, faculty deserve a rapid and powerful call for action that emphasizes the rethinking of the academic environment.

### 3.2. Student-Related Factors

Stress that results from teaching and student interaction is regarded as a main spoiler for the life satisfaction and well-being of university faculty members [92]. First, faculty at the entry level of their careers tend to be unprepared for professional teaching activities and proper student interaction, and investing time in these issues can lead to a decreased level of satisfaction [93]. Second, teaching and the associated preparation and evaluation of students through grading papers and providing feedback tends to be tedious and requires training and incentives. This leads not only to diminishing research time for faculty but also to the psychological frustration from feelings of inadequacy and being unable to derive benefits from the time they dedicate to their jobs [94,95]. The issue becomes more complicated when faculty have to handle large class sizes, putting them under an excessive work overload and mounting academic stress from being unable to devote sufficient time to their growing student cohorts [96].

The daily practice of teaching constantly forces faculty to deal with underprepared or struggling students who require extra layers of attention and support. The pressure to ensure student success and meet academic standards can create anxiety and cause faculty to feel discouraged about their work, with discomfort associated with low student performance [97]. Likewise, students who engage in disruptive behavior in the classroom, such as disrespect, cheating, plagiarism, or demand for additional resources or grades, can elicit strong emotions in educators and can impact their occupational well-being. Managing such disciplinary disturbances takes time and energy and can lead to frustration and stress [98,99,100]. Furthermore, prioritizing diversity among students has become widely adopted in higher education, and innovative approaches are used to augment the representation, comfort, and success of students from diverse populations. Improving the educational process to accommodate the needs of diverse students is challenging and adds anxiety and complexities to the teaching practices of faculty [101,102].

In all higher education institutions, the assessment of courses and programs is an internal process of quality control that engages students as stakeholders for the evaluation of faculty. Such evaluations commonly occur through the completion of anonymous paper-based or online questionnaires using rating scales such as the Likert scale [103,104]. Recent evidence points out that these universal student evaluations may have adverse consequences for the teaching staff, could contribute to occupational stress, and in some cases could be even insulting. During evaluation, the anonymity granted to students may allow them to use offensive comments, allegations, blame, and annotations on attire, appearance, and accent, all of which could be personally destructive and upsetting to faculty [105,106]. In a recent mixed-methods study conducted on Australian faculty, Lakeman and colleagues [107] reported significant distress among faculty from uncivil comments used in student evaluations. This was especially true for younger faculty and could affect recruitment, retention, and renewal of academic staff.

In an outlook on university students themselves, the literature indicates that they also struggle with course- and examination-related stress [108]. Some of them also experience lack of motivation, uncertainties over post-degree job security, and financial demands related to skyrocketing tuition costs [29]. For faculty to be able to undertake the emotional toll students impose on them, a focus on faculty well-being is crucial. In a recent study by Harding and colleagues [109], it was demonstrated that better teacher well-being is associated with better student well-being and fewer student difficulties. Student and faculty well-being interact, and reciprocal links exist between the psychology of the two populations, so supporting both will lead into more resilience, well-being, and fulfillment and to a better faculty–student relationship [110]. It is necessary for institutions to back up their faculty by providing necessary resources, professional development opportunities, and a reassuring work atmosphere to help mitigate student-related stressors.

### 3.3. Influence of the COVID-19 Pandemic

In December 2019, an outbreak caused by the severe acute respiratory distress syndrome coronavirus 2 (SARS-CoV-2) was spreading to different parts of the world, causing a respiratory illness with serious complications [111]. Soon thereafter, the associated disease, termed coronavirus disease 2019 (COVID-19), was declared a pandemic by the WHO [112]. Until the announcement that the pandemic was no longer a public health emergency in May 2023, COVID-19 cases accumulated to over 765 million, and the disease claimed approximately 7 million lives worldwide [113]. The pandemic effects have been shocking not only to patients but also to society, the environment, the global economy, and politics, leaving the world with an even worse financial crisis than that of 2008 [114,115]. Studies indicated a surge in mental health issues in the general population, as a consequence of the fear, anxiety, and isolation that accompanied the pandemic [116,117,118]. Education, much like other sectors of the society, has been profoundly affected, with an emergency shift to remote instruction, changes in the instructional tools and platforms, and modified assessment [119,120,121,122,123,124].

In the spring of 2020, higher education, heralded by the sudden and unprecedented manifestations brought about by the pandemic, underwent more substantial changes within a few weeks than it had in the previous ten years. As educators became aware of the breadth and complexity of the issues brought by the pandemic, improvements were frequently made and questions raised about procedures academia had in place for a long time for delivering educational programs. The outbreak scrambled the key assumptions and beliefs that serve as the foundation of higher education and altered the core components of place, time, and community that were previously utilized as the building blocks for universities’ success. Faculty, staff, and students had to quickly adjust to new circumstances, including how individuals teach and learn, how they collaborate with others, and how they juggle their personal and professional lives [125,126]. This along with the turbulence and uncertainty due to the pandemic did have its toll on the well-being of faculty, with reports of worrying symptoms of anxiety, depression, and stress [127,128,129,130], sometimes with younger individuals, the female gender, and persons with caring responsibilities experiencing the most severe manifestations [131]. In a systematic review and meta-analysis of mental issues due to COVID-19, it was shown that stress levels associated with the pandemic were more prevalent in university faculty compared to other educators such as school teachers. University faculty are responsible for training students in a variety of advanced, specialized skills and promoting the development of science and technology; they must also constantly interact with students, maintain a high level of professional performance, and meet targets and deadlines even in emergency times. As such, and during a period of uncertainty and high workload, such as the pandemic, stress among this group was high [132]. Faculty had to maintain their professionalism, constantly ensure quality, and modify courses, evaluations, extracurricular activities, student and committee services, research priorities, and various academic positions to fit the new COVID-19 frame. At the same time, they had to adjust to devalued compensation, budget cuts, research uncertainties, and the negative outcomes of remote work on their work–life balance and interaction with peers [133]. The literature has also introduced the notion of “techno stress” experienced by faculty due to the pandemic [134]. This is the pressure faculty face when they have to work online in a different, faster, and more demanding way, resulting in negative consequences on social, physiological, and psychological status; sleep disturbances; and social disruption, hence discouraging faculty from using technology [135]. Some factors such as low Internet bandwidth, lack of IT resources, crowded households, limited technological literacy, and lack of training in online teaching increased the burden on faculty and exacerbated psychological stress and mental health issues [136,137].

Nevertheless, promising data exist for faculty mental health changes after the pandemic, and positive insights indicate the flexibility and coping abilities of this resilient population. In a study of Polish faculty, post-pandemic levels of anxiety, stress, and depression significantly improved compared to initial levels reported at the beginning of COVID-19 and did not affect the academic performance of faculty [138]. Likewise, university professors from Jordan demonstrated good quality of life and mental health during COVID-19 lockdown, which were significantly associated with higher satisfaction with distance teaching, better health practices, and workload changes [139]. Accordingly, such factors underlying better well-being of faculty should be considered by academic institutions while they set up strategies for determining the best occupational structure in education and as a route for the improvement of the mental health of faculty and the preparedness for future pandemics.

Considering the impact of the pandemic on faculty mental health, it would be beneficial to explore how institutions can better support their faculty during times of crisis. This could involve the development of comprehensive mental health programs, promoting work–life balance, and fostering a sense of community, whereby not only subject matter expertise exists, but collaboration and communication are also fostered. Additionally, examining the effectiveness of the existing support systems and identifying areas for enhancement can contribute to creating a healthier, more resilient academic environment. The lessons learned from COVID-19 envision an upscaling of higher education institutions into inclusive, learner-centered, flexible organizations where various educational frameworks can be leveraged, and faculty are properly trained in both pedagological and technological domains [140]. Another important lesson learned from the pandemic relates clearly to mental health. Given the pandemic-related multiple stressors and traumatic events, a stress- and trauma-informed mental health response became crucial. As such, an emerging governance model of the pandemic promotes a suitable environment for the implementation of public mental health interventions, normalizes support seeking, and reduces stigma [141]. A reflection by universities on the faculty experiences in mental health faced in the wake of the global pandemic can provide valuable insights into mental health areas that need focus and improvement.

### 3.4. Changes in Higher Education and Research Structures

Across many countries, higher education has been influenced by a progressively competitive discourse over the past three decades, with the number of enrolled students increasing by about three fold and with emphasis on the efficiency, accountability, and marketization of education [142,143]. Such massification in undergraduate education has led to an environment where competition among institutions and university branding have flourished [65]. Students have been reframed and repositioned as customers whose needs should be catered to by universities and who should benefit from an excellent and rewarding student experience [144,145]. This consumerism perspective results in the unreasonable expectations by students that paying for their education entitles them to certain grades, achievements, benefits, and preferential treatment in terms of courses or grades without investing significant time or effort [146,147]. This emerging aspect of academic entitlement has serious educational consequences among current university generations known to be tolerant, narcissistic, confident, and ungratifying [148]. Jiang and colleagues [149] demonstrated that such instructor-reported uncivil behaviors of students are related to faculty burnout and emotional exhaustion, raising questions on teaching effectiveness. Similar findings regarding academic entitlement and decreased faculty well-being were reported elsewhere [150,151], indicating that the conflict between university structures favoring consumer retention and satisfaction and classroom expectations by faculty can lead to dissatisfaction and anxiety [29]. Moreover, the profound changes experienced by universities, such as globalization and mobility, greater institutional autonomy, changes in governance and management, demanding institutional deliverables, changes in recruitment patterns with preference for short-term contracts, and increased administrative duties, have resulted in calamitous consequences on academic professionalism, academic freedom, and job security [152,153]. Universities consistently generate administrative, pedagogical, and technological demands that are often overwhelming and exert a psychological and physical toll that affects the sustainability and collegiality of academics [65].

Like in education, the competitive perspective has also cascaded into research. Academic institutions have embraced the importance of transferring scientific and technological knowledge into production and diffusion, with wide agreement on the need for the propagation of market-oriented concepts and practices [154,155]. This has resulted in unequal distribution of funds towards academics in Science, Technology, Engineering, and Math (STEM) fields at the expense of those in social sciences and humanities, perhaps creating personal conflicts for the latter group [156]. In general, universities are often more interested in the research findings and success stories of their faculty members than in their personal stories or mental well-being. Despite the culture of assessment and evaluation, it is uncommon for universities to conduct surveys that explore their faculty’s satisfaction and mental health. Accordingly, handling stress and anxiety and having the durability to mental health challenges are not only part of the faculty career path but are also key criteria for whether a person appears fit for a job in academia [63].

## 4. Review of the Literature Addressing Mental Health in Academics

Compared to the expanded efforts in exploring mental health among university students [157,158,159,160,161] and some workforce categories, especially healthcare personnel [162,163,164,165], the literature addressing mental health in university faculty remains limited. In the following sections, a summary of the principle mental health issues of faculty, namely anxiety, depression, stress, and burnout, is presented. The common scales used for measurement are described, and a snapshot of some pertinent studies is captured in Table 1. Although the data in Table 1 show four mental health conditions and their prevalence in faculty across different countries using variable tools, the heterogeneity of data in Table 1 and the paucity of evidence collected both call for large, inclusive studies on faculty well-being. Such studies should use validated, standardized tools and representative academic populations to obtain a better portrait of anxiety, depression, stress, and burnout among faculty.

### 4.1. Studies Addressing Anxiety and Depression

Anxiety and depression are amongst the most disabling mental health conditions, and both are ranked among the top 25 leading causes of disease burden worldwide [178]. According to the WHO, anxiety conditions are characterized by excessive fear, worry, and related behavioral disturbances. Symptoms of anxiety can be severe enough to result in significant distress or significant impairment of functioning [179]. The global prevalence anxiety is around 3.6% [180], and it is more prevalent in urbanized countries [181]. Depression is another common mental disorder that involves a depressed mood or loss of pleasure or interest in activities for long periods of time. Depression can affect all aspects of life, including relationships with family, friends, and community. An estimated 3.8% of the population experiences depression, including 5% of adults (4% among men and 6% among women) and 5.7% of individuals older than 60 years. In the first year of the COVID-19 pandemic, the global prevalence of anxiety and depression increased by a massive 25% [182].

Among faculty, depression and anxiety were somehow linked to age below 40 years and female gender [166,167]. Studies exploring the prevalence of anxiety and depression commonly use the combined Depression, Anxiety, and Stress Scale—21 Items (DASS-21) [183], which is a validated tool that can indicate the prevalence of the three mental issues simultaneously. Longer and shorter versions of the DASS-21, including 42, 12, or 8 items, respectively, have been used in various studies [184,185,186] but rarely in faculty. Additional scales used include the Beck’s Anxiety Inventory (BAI) and Beck’s Depression Inventory (BDI) [187] and others, as shown in Table 1. It remains tempting to speculate observations of anxiety and depression among university faculty and check the utility of various interventions, as has been done for other populations [188,189,190].

### 4.2. Studies Addressing Stress

Stress represents an integral part of human life and is perhaps one of the most common issues in modern societies [191]. According to the WHO, stress is defined as a state of worry or mental tension caused by difficult situations. While stress is a natural human response that allows to address challenges and threats, excess stress can precipitate physical and mental health issues and affect overall well-being [192]. Occupational stress is one of the most significant issues for workers, where long hours and job strain are two common workplace stresses that have a negative impact on most demographic groups. These stressors have been linked to major causes of morbidity such as cardiovascular disease, diabetes, and depression [193]. Furthermore, employees who experience work stress have an average 50% increased risk of coronary heart disease [194].

The DASS-21 tool and other measures were used in studies to detect stress among faculty members, as shown in Table 1. Additionally, the Faculty Stress Index (FSI) developed by Gmelch, Wilke, and Lovrich [195] consists of 45 survey items that ensure all potentially relevant aspects of job-related stress are adequately explored. The items are subdivided into five factors: (1) reward and recognition, which pertain to rewarding expectations for community and university services, financial needs, and insufficient recognition; (2) time constraints, which confront the faculty member on general duties such as paperwork, meetings, phone and visitor interruptions, and sufficient time for professional development, teaching, and service; (3) departmental influence, which focuses on the extent to which faculty perceive their department as controlling over their work or the level of autonomy that faculty members feel they have within their departmental facets, such as resolving differences, knowing evaluative criteria, and influencing decisions at departmental/institutional levels; (4) professional identity, referring to reputation as a scholar established on the basis of publications, presentations at professional meetings, and successful securing of research grants; and (5) student interaction, which considers student instruction in the classroom, evaluation, and advising. In a study using FSI to evaluate stress among business faculty, class presentations and criteria for evaluating research contributed to the least stress levels, while heavy workload generated the highest level of stress [60]. Other findings reported that student interaction followed by professional identity factors were highly involved in faculty stress among participants from management, finance, science, and language departments, thus calling for focus on these areas through regular communication and support [196]. In another investigation, faculty in three different institutions who practiced a healthy/spirituality regimen significantly reduced stress in areas of time constraint, reward and recognition, and departmental influence, indicating the need to seek effective means of faculty stress reduction [197]. Similarly, and although using a different measure of stress, student factors and lack of recognition were the most significant stressors among dental faculty [198].

It is noteworthy that stress levels among faculty were affected by COVID-19, much like other professionals [199,200]. For instance, Garcia and colleagues [201], in their longitudinal analysis of work-related stress among faculty before and during COVID-19, demonstrated a negative impact of the pandemic on work overload, powerlessness, and poor peer relationships, suggesting that these constructs may influence mental health and work productivity. As such, workload reduction, empowerment, and collegial activities may be areas for administrators to contemplate in order to help ease the burden brought about by pandemic and generally alleviate stress for faculty members. In light of the aforementioned areas of stress and others that may exist in particular national contexts [202,203], policymakers should effectively design guidelines to minimize stress at the personal and organizational levels and ensure the availability of convenient resources that lessen stress among faculty.

### 4.3. Studies Addressing Burnout

The concept of burnout was first described by Freudenberger in 1974 to include three main aspects: exhaustion, expressed as the lack energy even after rest; depersonalization, indicating disconnection from others and lack of interest in important things; and feelings of ineffectiveness even after achievement of a good outcome [204]. Although such features of burnout are somehow similar to major depression or, in some cases, to severe anxiety, burnout tends to occur mostly due to chronic stress in an occupational environment or in the workplace [205]. Nowadays, burnout has become one of the most important occupational hazards, causing significant costs for both individuals and organizations [206]. Although burnout was initially considered a particular issue of professionals working in patient care, it is now known that this syndrome can develop among all professions [207,208,209,210]. Given the enormous negative impact of burnout on the careers and personal lives of workers as well as on the economy and public health of society at large, the WHO included this syndrome in the 11th Revision of the International Classification of Diseases (ICD-11) as a phenomenon exclusive to the occupational context [211].

Few studies addressing burnout among faculty used the Maslach Burnout Inventory (MBI) [212], although other measures, some of which are listed in Table 1, were used as well. The MBI measures three dimensions of burnout: (1) emotional exhaustion, which comprises feelings and sensations of being exhausted by the psychological efforts associated with work; (2) depersonalization, a response of detachment, indifference, and lack of concern towards the work performed and/or the people receiving it, with negative attitudes of irritability and avoidance; and (3) inefficacy, a negative professional self-evaluation and doubt about the ability to perform a job effectively, with a decrease in productivity, capabilities, and morale [211]. MBI is currently the gold standard for measuring burnout in medical research [177,213,214]. Studies utilizing the MBI identify high levels of emotional exhaustion, depersonalization, and reduced personal efficacy among faculty members. The findings indicate that the unique and demanding nature of academic work contributes to burnout in this population [177,215]. Because academics may be particularly susceptible to burnout and its professional and personal implications, the need for proactive strategies and support systems to address burnout and promote the well-being of university faculty should be emphasized.

### 4.4. Focused Insight into Mental Health of Faculty Working in Health Education

Research and education stand at the forefront of modern healthcare and are roles largely played by faculty. The field of medicine needs savvy, devoted clinician–scientists who can persevere with one foot in clinical practice and another in the research arena, thus playing a special role in improving patient care through investigation and mentoring [216]. Nevertheless, evidence shows that faculty who are practitioners–academics or who work in health education face high levels of stress, burnout, and depression [62,217]. Due to the inherent characteristics of their work, healthcare professionals, especially those who work in hospital settings, are at risk of emotional stress, negative mental consequences, and burnout, causing challenges that affect their daily practice and the quality of care [218,219]. Despite providing excellent care to patients, healthcare professionals frequently struggle with setting a priority for their own self-care [217]. As such, nurses, physicians, pharmacists, and other healthcare professionals are at higher risk for having mental health issues when compared to the general adult population [220,221,222,223], and this was even worsened by the COVID-19 pandemic [224,225]. Limited resources, occupational hazards affecting one self and family, longer working shifts, increased workload, sleep disruption, and the rampant avalanche of information have all contributed to healthcare professionals’ increased physical and mental fatigue, anxiety, stress, and burnout during the pandemic [226,227]. All such attributes, if added to the nature of a career in academia, demonstrate the significance of addressing the mental health of faculty who are involved in health education as a particular faculty category with dual responsibilities, both of which are demanding and require high competence.

Evidence shows that burnout is particularly significant among faculty members in pharmacy, medicine, and nursing due to the need for these professionals to balance several high-stake tasks. These include teaching, research, community engagement, and administrative work, all on top of providing appropriate patient care [177,228]. In this regard, the attention to the mental health of faculty/healthcare professionals becomes ultimately important in light of research indicating its direct influence on patients. Evidence exists that when the healthcare staff’s wellness is not optimal, the performance of healthcare systems is less efficacious, and medical errors significantly increase [229,230]. Numerous expectations exist of healthcare professionals who are also academics: firstly from their patients, who anticipate professionalism, skills, and compassion, and secondly from superiors and employers, who expect from these faculty a full teaching load, research productivity, escalating patient numbers, and revenues. For some professionals, these expectations lead to increased productivity; meanwhile, for others, such overloading of duties may lead to dissatisfaction, job distress, and desires to leave the profession [231,232]. Examples of studies that show major mental health findings and some associated factors in health faculty/practitioners from different healthcare professions are presented below in Table 2. If mental health issues are reported in faculty and also in healthcare practitioners, it is relevant to direct focused attention to those who practice both roles, with the purpose of maintenance of their well-being, which can influence both healthcare and education. The systematic consolidation of evidence from studies summarized in Table 2 and similar investigations shall provide a critical background in designing strategies to reduce stress and burnout in health-education faculty and increase their retention, productivity, and job satisfaction.

## 5. Strategies to Promote Mental Well-Being among Academics

Mental health among faculty is not an individual concern but rather a responsibility of all the critical components of the academic career, which hold different accountabilities of perpetuating policies, practices, and behavior norms that affect well-being [29]. As such, a systematic and proactive approach involving faculty, institutions, and culture is urgently needed to instigate a fundamental change that prioritizes the well-being of academics. In public mental health, the WHO European framework for action on mental health 2021–2025 [238], which was primarily launched as a part of the COVID-19 response and recovery, provides a coherent basis for intensified efforts to safeguard mental well-being. The main themes of this framework revolve around integration of mental health in universal health coverage; ensuring promotion of mental health over the life course; and implementation of these themes through leadership, advocacy, and data availability. In higher education, the evidence of the effectiveness of strategies to promote mental health remains inconclusive and mostly focused on students [239]. Although initiatives to promote well-being are multiple and broad, they still fall short of cultivating faculty mental health, and a multi-layer approach similar to the WHO framework or protracted from its main concepts remains necessary [65]. Should universities consider the mental health of faculty a priority, investment in physical and human resources for this purpose needs to be put forward and profit from several initiatives in this regard that have been previously set in the governmental [240,241,242], health [243,244,245], and business [246,247] sectors. An overview of suggested strategies for overall mental health improvement in academics as built on planning, institutional capacity, and faculty role is presented below.

### 5.1. Building an Organizational Plan That Prioritizes Mental Health in Academia

The influence of organizational culture on faculty well-being cannot be underestimated, as the psychological workplace environment is intricately connected to individual mental health [248]. Therefore, workplaces can be sources of well-being [249], and a supportive workplace environment is associated with diminished levels of job dissatisfaction, burnout, and depression [250]. According to earlier research, interventions in mental health that adopt an organizational approach, whereby action starts at the management level, may have significant positive effects on mitigating burnout [251,252], reducing anxiety [253], and alleviating feelings of stress [254]. At universities, strategies to support the mental health of faculty should start with a thorough evaluation and research of the present circumstances and diligently look into factors associated with faculty stress and burnout. Once these factors are identified and acknowledged, a commitment to meet faculty mental health needs should be made [62]. Such a commitment should uphold the whole university community at its core, considering the resources, needs, and demands of all stakeholders among both students and faculty to establish a sustainable, flexible background that molds the university towards cultivating a mentally healthy work environment [65]. Nevertheless, social stigma, research limitations, reduced access to mental health services, resource shortages, and cost-effectiveness remain key challenges associated with the implementation of major, institution-wide mental health strategies [255].

Universities can adopt numerous plans to optimize their support for faculty, namely reevaluation of workflow, reasonable allocation of responsibilities, capacity building of support staff to maximize efficiency, and investing in wellness programs [61]. When faculty are actively engaged in the planning and implementation of mental health strategies, their sense of ownership can improve acceptability and success [256]. Faculty ownership can be fostered through inclusive and participatory processes in the mental health strategy, where faculty should be involved in the development and refinement of the guidelines, ensuring that their perspectives and experiences are heard and valued. In addition, creating opportunities for faculty engagement, such as establishing cross-disciplinary committees or task forces, possibly also with student and staff representation, can empower faculty to contribute actively and exhibit a sense of collective responsibility towards well-being. Moreover, mental health strategies should be aligned with existing organizational objectives and prioritized. The existing work practices and mindsets should be slowly tuned, while new, more promising ones are adapted with ongoing observation, evaluation, and reflection. This shall maximize both the practical and scientific impact of a mental health plan and allow its transfer beyond the specific organization [257].

Interestingly, some universities have set some exemplars for spearheading a positive change in wellness perspectives that affect faculty. For instance, the “Mentally Healthy Universities Program” was a pilot project conducted over nine universities in the U.K. to improve the mental health and well-being of both faculty and students. In this program, running from 2019 until 2021, six primary goals were set: (1) prioritize mental health in the workplace by developing and delivering a systematic program; (2) proactively ensure that organizational culture drives positive mental health outcomes; (3) promote an open culture around mental health; (4) increase organizational confidence and capability; (5) provide mental health tools and support; and (6) increase transparency and accountability through internal and external reporting. The piloting evaluation showed the positive experience of students and staff, reporting increases in confidence, understanding, and awareness towards mental health [258].

### 5.2. Institutional and Leadership Support

Institutions can adopt many strategies to support their faculty and mitigate faculty burnout, stress, and emotional issues. Leaders, through servant leadership and modeling by example, can moderate extreme and competitive environments and improve collegiality, teamwork, and collaboration [61,259]. Institutional approaches to demonstrate support for faculty include providing resources for professional development in teaching, research, and service as well as appreciation, encouragement, and reassuring faculty of their value. Furthermore, fair distribution of tasks, transparency of tenure and promotion expectations, equitable appraisal and rewards, and timely, effective, inclusive communication and a leadership that embeds diversity and equity are all of the utmost importance [260]. A working environment in which gratitude is spread reassures growth, minimizes negative emotions, buffers exhaustion, and generally renders the workplace more pleasant and productive [261,262].

The importance of mentoring as a strategy for mental health prevention and promotion of the quality of life has been previously documented [263,264]. In academia, the importance of mentorship was also corroborated in research by El-Ibiary and colleagues, who proved higher burnout in faculty without a mentor [177]. As academics progress in their career, their success becomes more tough to define since they receive less feedback as they become senior [54]. Nevertheless, the beneficial effects of mentorship that potential mentees and effective mentors experience cannot be overlooked. Mentoring enhances productivity, efficiency, and motivation, and formal mentoring programs have an overall positive impact on junior faculty who are taken under the wings of mentors for guidance and motivation [265,266]. Moreover, mentorship at various academic departments is key to career satisfaction and retention, as reported previously [267]. Hence, universities should give more attention and consideration to mentorship and should acknowledge that faculty may be struggling due to a dearth of formal and/or informal support from their leadership. New faculty may be truly in need for development, but areas of new technology, pedagogy, and scholarship do not stop for faculty, even those at an advanced career stage. Faculty empowerment to support each other through mentoring strengthens individual faculty members, fortifies the institution, reduces faculty burnout, and nurtures job satisfaction [268]. A systematic investigation of the specific effects of faculty mentorship programs on mental health remains an interesting area to explore.

Apart from faculty development and mentoring, institutions can offer a wide range of support structures for faculty. For example, openness to work flexibility balances life and work for the academic staff and mitigates work-related stress [61]. In 2022, Shiri and colleagues [269], in their systematic review of flexible work, concluded that it has small beneficial effects on mental health and called for thorough investigation in this regard. Flexibility is reported among the key factors for job engagement and mental well-being [270], for instance, through a shorter working week, flexible schedules, and telecommunication, all of which increase autonomy over personal workload and reduce burnout [271]. Another modifiable cause of faculty stress is email communication [272]. Overdependence on emails, a reality commonly encountered in academic settings, results in feelings of email overload and stress probably due to longer working hours and blurred work–life boundaries [273]. The academic leadership’s setting of email expectations can promote wellness through scheduling business time in contrast to downtime without electronic interruptions, and this was reported to reduce stress [274]. Likewise, frequent meetings are known to reduce work-related resources if they consume too much time, lack structure, and are nonproductive [275]. Academic leaders should plan and lead effective meetings b defining meeting purposes, developing of detailed agendas, inviting critical participants, determining the suitable meeting format, and communicating necessary information and supporting documents in advance [276]. Leaders should be aware that while some meetings are important and necessary, others can be substituted by mails or executive-level decisions, thus sparing faculty the time and effort needed and supporting them in terms of well-being and productivity [61].

When drafting policies, academic leaders at all levels should pause and check the impact of their policies, actions, and leadership style on faculty. When assigning new tasks and responsibilities, leaders should take the time to reconcile with their staff, considering not only their current career priorities but also their personal circumstances and well-being. Such discussions will significantly ameliorate faculty members’ stress levels and convey the idea that both their achievements and their well-being are equally important [63].

### 5.3. Individual Strategies

Despite the importance of strategic vision and leadership initiatives in promoting faculty well-being, faculty are also personally responsible for adopting habits that reduce their own stress, make them more productive, and reduce the assault on their personal privacy with work demands [277]. In their pursuit of work–life balance, in seeking support whenever needed, and in advocacy for a change in academic culture, faculty must be proactive and responsible. First and foremost, various evidence-based lifestyle modifications should be implemented, including healthy diet, hydration, regular exercise, sufficient sleep, quitting smoking, and maintaining a healthy weight [278]. The WHO guideline on self-care interventions for health and well-being [279] provides a holistic approach for every day wellness that is rooted in evidence-based recommendations and good-practice statements. It can be used to personalize faculty approaches to well-being and cater to their diverse needs based on individual priorities.

Time-management and self-organization skills are another cornerstone of productivity, effective self-realization, ability to compete, and personal success. As such, these attributes promote mental health [280] and should be considered to reduce the stress and anxiety that arise from the multiple commitments of faculty [29]. Balancing life with career means that faculty are able to set realistic expectations of their workload and are also able to advocate for a culture of declining additional responsibilities and opportunities in favor of making time for themselves, their family, and their research [54]. Setting boundaries applies to working with students, delegating assignments, distributing the workload, cutting additional tasks, and using smart time-management approaches. This gives the opportunity to relax, unwind, and disengage from work in an era where work emails are accessible by smartphones, making everyone constantly available and reachable. Recent evidence suggests that checking emails less frequently alleviates stress and perpetuates numerous measures of well-being [274].

Seeking support in the context of academic settings can be quite helpful for faculty. Research shows that social support can mediate a decrease in feelings of depression and anxiety [281,282,283]. Hence, building social networks and reducing loneliness along the academic path is vital and effective [284]; it can improve work dynamics and help faculty to persist in a demanding environment through building collaborations and team work [285]. Moreover, faculty must recognize that reaching out to others for mental health support is essential, whether it is colleagues in the workplace or professional counselors. The stigma associated with mental health problems leaves individuals feeling that they have to hide their difficulties, and this can be more devastating than the mental issues themselves. The support in making disclosure decisions can potentially improve well-being, reduce stigma, and enhance recovery [286,287].

## 6. Conclusions and Future Directions

Thus far, the literature has highlighted the mental health and well-being issues affecting all levels of the academic hierarchy, from early-career faculty to those in senior placements. Depression, anxiety, stress, burnout, isolation, lack of support, a normalized philosophy of overwork, and a competitive culture are all experiences that appear linked to a career in university education. Increasing internal and external pressures from university structures, student numbers, attitudes and diversity, and research burden are some of the factors behind the common frustration that impedes the wellness of professionals in the academic field. A collaborative effort that is initiated at the level of organizational planning and strategic initiatives streamlined to leadership and utilized by faculty to foster their well-being should be advocated.

Major efforts are still required in terms of establishing clear frameworks for the maintenance of the well-being of faculty and evidence-based understanding of the factors, consequences, and implications associated with their mental health. Not only does qualitative and quantitative investigation in this field remain tempting and much-needed, but an outlook into advanced technological tools such as digital mental health interventions [288] as well as artificial-intelligence-guided therapy and life-crafting [289] is also important. These and other tools may show promise within the larger frame of strategies, actions, and assessments that higher education institutions need to implement to foster the mental health of faculty. Exploring the feasibility and evaluating the effectiveness of these interventions, including digital technology, remains an interesting area in which research and practice capacities need to be built in order to obtain evidence regarding mental health outcomes. A higher education institution should be an environment where faculty develop, learn, teach, and thrive without compromising their well-being. The transformation of universities into genuinely healthy settings requires a key change in the working culture where creative, unbiased strategies that are deeply rooted in firm research that promotes mental health should be applied.

## Figures and Tables

**Table 1:** Examples of studies assessing anxiety, depression, stress, and burnout among faculty, with country, scale used, and prevalence of the mental condition.

Mental Health Issue	Country	Scale Used	Prevalence	Reference
Anxiety	USA	DASS-21	39%	[33]
	Malaysia	DASS-21	50%	[166]
	KSA	BAI	12%	[167]
	USA	Generalized Anxiety Disorder—2-item Scale	12%	[168]
Depression	USA	DASS-21	38%	[33]
	Malaysia	DASS-21	29%	[166]
	KSA	BDI	67%	[167]
	USA	BDI	12%	[169]
	USA	Epidemiologic Studies Depression Scale	5%	[170]
	USA	Patient health questionnaire	13%	[168]
Stress	USA	DASS-21	31	[33]
	Malaysia	DASS-21	15%	[166]
	India	Job stress questionnaire	23%	[171]
	USA	Perceived Stress Scale	33%	[172]
	USA	Perceived Stress Scale	34%	[173]
	Jordan	Kessler Distress Scale	70%	[129]
	Ethiopia	University and College Union stress questionnaire	60%	[174]
Burnout ^1^	Korea	MBI	34%	[175]
	USA	MBI	38%	[176]
	USA	MBI	10%	[177]
	USA	Non-proprietary single-item measure	35%	[168]

DASS-21, Depression, Anxiety, and Stress Scale—21 Items; BAI, Beck’s Anxiety Inventory; BDI, Beck’s Depression Inventory; MBI, Maslach Burnout Inventory. ^1^ Prevalence rates of burnout are reported in terms of minimum rates observed for any of the three dimensions of the MBI.

**Table 2:** Examples of studies tackling the mental health issues of particular health-education faculty.

Health Profession	Specific Faculty Groups within the Profession	Key Mental Health Findings from Sample Studies	Reference
Physicians	Medical and surgical faculty	23% rate of extreme stress; 16–23% rate of burnout	[171]
	Clinical and non-clinical faculty in a teaching hospital	31% rate of high job stress, associated with work in a clinical department; 30–88% rate of burnout	[233]
	Chairs of obstetrics and gynecology departments	Over 50% rates of low personal accomplishment	[234]
	Academic physicians in a university hospital	43% rate of distress; 78% of distressed physicians did not seek professional help	[235]
Pharmacists	Pharmacy practice faculty	41% rate of emotional exhaustion, significant in women and assistant professors	[177]
	Academic pharmacists in a national survey	Better work–life balance significantly associated with intention to stay in academia	[236]
Nurses	Nursing faculty	Full-time employment, working over 45 h per week, and teaching 3–4 courses correlated with high burnout	[237]
	Nursing faculty	27% rate of depression; better workplace culture was associated with less depression, anxiety, and burnout	[168]

## Data Availability

Data sharing is not applicable to this article.

## References

[1] Murkatik K., Harapan E., Wardiah D. (2020). The influence of professional and pedagogic competence on teacher’s performance. J. Soc. Work Sci. Educ..

[2] Umbach P.D., Wawrzynski M.R. (2005). Faculty do matter: The role of college faculty in student learning and engagement. Res. High. Educ..

[3] Melnyk B.M. (2023). Fixing broken systems and unhealthy cultures in healthcare and educational institutions is key to improving the mental health and well-being of nurses, the healthcare workforce, faculty, and students. Worldviews Evid. Based Nurs..

[4] Martineau M., Beauchamp G., Marcotte D. (2017). Efficacy of mental health prevention and promotion strategies in higher education. Sante Ment. Que..

[5] Mari J., Galea S. (2021). Editorial: Cities and population mental health: Present and future. Curr. Opin. Psychiatry.

[6] GBD 2019 Mental Disorders Collaborators (2022). Global, regional, and national burden of 12 mental disorders in 204 countries and territories, 1990–2019: A systematic analysis for the Global Burden of Disease Study 2019. Lancet Psychiatry.

[7] Strengthening Mental Health Promotion Fact sheet no. 220. World Health Organization: Geneva, Switzerland, 2022. https://www.who.int/en/news-room/fact-sheets/detail/mental-health-strengthening-our-response.

[8] Centers for Disease Control and Prevention (2023). About Mental Health. https://www.cdc.gov/mentalhealth/learn/index.htm#:~:text=Mental%20health%20includes%20our%20emotional,childhood%20and%20adolescence%20through%20adulthood.

[9] (2015). Chronic Illness & Mental Health. Bethesda, MD: National Institutes of Health, National Institute of Mental Health. https://www.nimh.nih.gov/health/publications/chronic-illness-mental-health.

[10] Sharpe M. (2020). Time to transcend “physical” and “mental” health. BMJ.

[11] (2022). World Mental Health Report: Transforming Mental Health for All.

[12] Das K., Pingali M.S., Paital B., Panda F., Pati S.G., Singh A., Varadwaj P.K., Samanta S.K. (2021). A detailed review of the outbreak of COVID-19. Front. Biosci..

[13] Filipčić I., Isaac M. (2022). Editorial: The mental health in post-COVID-19 era: Challenges and consequences. Curr. Opin. Psychiatry.

[14] Bourmistrova N.W., Solomon T., Braude P., Strawbridge R., Carter B. (2022). Long-term effects of COVID-19 on mental health: A systematic review. J. Affect. Disord..

[15] Weich S. (2022). Mental health after covid-19. BMJ.

[16] Jin Y., Sun T., Zheng P., An J. (2021). Mass quarantine and mental health during COVID-19: A meta-analysis. J. Affect. Disord..

[17] Fancourt D., Steptoe A., Bu F. (2021). Trajectories of anxiety and depressive symptoms during enforced isolation due to COVID-19 in England: A longitudinal observational study. Lancet Psychiatry.

[18] (2022). COVID-19 Mental Health and Wellbeing Surveillance: Report. https://www.gov.uk/government/publications/covid-19-mental-health-and-wellbeing-surveillance-report.

[19] Lannin D.G., Vogel D.L., Brenner R.E., Abraham W.T., Heath P.J. (2016). Does self-stigma reduce the probability of seeking mental health information?. J. Couns. Psychol..

[20] Zweifel P. (2021). Mental health: The burden of social stigma. Int. J. Health Plan. Manag..

[21] Tong A.C.Y., Tsoi E.W.S., Mak W.W.S. (2021). Socioeconomic Status, Mental Health, and Workplace Determinants among Working Adults in Hong Kong: A Latent Class Analysis. Int. J. Environ. Res. Public Health.

[22] Bartoll-Roca X., Palència L., Gotsens M., Borrell C. (2022). Socioeconomic inequalities in self-assessed health and mental health in Barcelona, 2001-2016. Gac. Sanit..

[23] Thompson G., McBride R.B., Hosford C.C., Halaas G. (2016). Resilience Among Medical Students: The Role of Coping Style and Social Support. Teach. Learn. Med..

[24] Dubreucq J., Plasse J., Franck N. (2021). Self-stigma in Serious Mental Illness: A Systematic Review of Frequency, Correlates, and Consequences. Schizophr. Bull..

[25] Henderson C., Gronholm P.C. (2018). Mental Health Related Stigma as a ‘Wicked Problem’: The Need to Address Stigma and Consider the Consequences. Int. J. Environ. Res. Public Health.

[26] The Lancet Global Health (2020). Mental health matters. Lancet Glob. Health.

[27] (2018). Mental Health Atlas 2017.

[28] Doran C.M., Kinchin I. (2019). A review of the economic impact of mental illness. Aust. Health Rev..

[29] Johnson A.P., Lester R.J. (2022). Mental health in academia: Hacks for cultivating and sustaining wellbeing. Am. J. Hum. Biol..

[30] Hill N.T.M., Bailey E., Benson R., Cully G., Kirtley O.J., Purcell R., Rice S., Robinson J., Walton C.C. (2022). Researching the researchers: Psychological distress and psychosocial stressors according to career stage in mental health researchers. BMC Psychol..

[31] Lipson S.K., Eisenberg D. (2018). Mental health and academic attitudes and expectations in university populations: Results from the healthy minds study. J. Ment. Health.

[32] Abelson S., Lipson S.K., Eisenberg D., Perna L.W. (2022). Mental Health in College Populations: A Multidisciplinary Review of What Works, Evidence Gaps, and Paths Forward. Higher Education: Handbook of Theory and Research.

[33] Meeks K., Peak A.S., Dreihaus A. (2023). Depression, anxiety, and stress among students, faculty, and staff. J. Am. Coll. Health.

[34] Evans T.M., Bira L., Gastelum J.B., Weiss L.T., Vanderford N.L. (2018). Evidence for a mental health crisis in graduate education. Nat. Biotechnol..

[35] Sheldon E., Simmonds-Buckley M., Bone C., Mascarenhas T., Chan N., Wincott M., Gleeson H., Sow K., Hind D., Barkham M. (2021). Prevalence and risk factors for mental health problems in university undergraduate students: A systematic review with meta-analysis. J. Affect. Disord..

[36] Chang J.J., Ji Y., Li Y.H., Pan H.F., Su P.Y. (2021). Prevalence of anxiety symptom and depressive symptom among college students during COVID-19 pandemic: A meta-analysis. J. Affect. Disord..

[37] Li W., Zhao Z., Chen D., Peng Y., Lu Z. (2022). Prevalence and associated factors of depression and anxiety symptoms among college students: A systematic review and meta-analysis. J. Child Psychol. Psychiatry.

[38] Auerbach R.P., Alonso J., Axinn W.G., Cuijpers P., Ebert D.D., Green J.G., Hwang I., Kessler R.C., Liu H., Mortier P. (2016). Mental disorders among college students in the World Health Organization World Mental Health Surveys. Psychol. Med..

[39] Okoro C., Owojori O.M., Umeokafor N. (2022). The Developmental Trajectory of a Decade of Research on Mental Health and Well-Being amongst Graduate Students: A Bibliometric Analysis. Int. J. Environ. Res. Public Health.

[40] Woolston C. (2019). PhDs: The tortuous truth. Nature.

[41] Woolston C. (2020). Signs of depression and anxiety soar among US graduate students during pandemic. Nature.

[42] Wang X., Hegde S., Son C., Keller B., Smith A., Sasangohar F. (2020). Investigating Mental Health of US College Students During the COVID-19 Pandemic: Cross-Sectional Survey Study. J. Med. Internet Res..

[43] Walters M., Alonge T., Zeller M. (2022). Impact of COVID-19 on Medical Education: Perspectives From Students. Acad. Med..

[44] Wilson O.W.A., Holland K.E., Elliott L.D., Duffey M., Bopp M. (2021). The Impact of the COVID-19 Pandemic on US College Students’ Physical Activity and Mental Health. J. Phys. Act. Health.

[45] Zapata-Ospina J.P., Patiño-Lugo D.F., Marcela Vélez C., Campos-Ortiz S., Madrid-Martínez P., Pemberthy-Quintero S., Pérez-Gutiérrez A.M., Ramírez-Pérez P.A., Vélez-Marín V.M. (2021). Mental health interventions for college and university students during the COVID-19 pandemic: A critical synthesis of the literature. Rev. Colomb. Psiquiatr. (Engl. Ed.).

[46] Holm-Hadulla R.M., Klimov M., Juche T., Möltner A., Herpertz S.C. (2021). Well-Being and Mental Health of Students during the COVID-19 Pandemic. Psychopathology.

[47] Elmer T., Mepham K., Stadtfeld C. (2020). Students under lockdown: Comparisons of students’ social networks and mental health before and during the COVID-19 crisis in Switzerland. PLoS ONE.

[48] Copeland W.E., McGinnis E., Bai Y., Adams Z., Nardone H., Devadanam V., Rettew J., Hudziak J.J. (2021). Impact of COVID-19 Pandemic on College Student Mental Health and Wellness. J. Am. Acad. Child Adolesc. Psychiatry.

[49] Müller C., El-Ansari K., El Ansari W. (2022). Cross-Sectional Analysis of Mental Health among University Students: Do Sex and Academic Level Matter?. Int. J. Environ. Res. Public Health.

[50] Mahdavi P., Valibeygi A., Moradi M., Sadeghi S. (2023). Relationship Between Achievement Motivation, Mental Health and Academic Success in University Students. Community Health Equity Res Policy.

[51] Borges L.O., Motta G.M.V., Garcia-Primo G.M., Barros S.C., Heleno C.T. (2023). Working Conditions and Mental Health in a Brazilian University. Int. J. Environ. Res. Public Health.

[52] Tessarini Junior G., Saltorato P. (2021). Organização do trabalho dos servidores técnico-administrativos em uma instituição federal de ensino: Uma abordagem sobre carreira, tarefas e relações interpessoais. Cad. EBAPE. BR.

[53] Harris B.R., Maher B.M., Wentworth L. (2022). Optimizing Efforts to Promote Mental Health on College and University Campuses: Recommendations to Facilitate Usage of Services, Resources, and Supports. J. Behav. Health Serv. Res..

[54] Lashuel H.A. (2020). What about faculty?. eLife.

[55] Smith J.M., Smith J., McLuckie A., Szeto A.C.H., Choate P., Birks L.K., Burns V.F., Bright K.S. (2022). Exploring Mental Health and Well-Being Among University Faculty Members: A Qualitative Study. J. Psychosoc. Nurs. Ment. Health Serv..

[56] Carvajal R.F.P., Guedea M.T.D. (2021). Stress in university research professors: A systematic review. Salud Ment..

[57] Mainous A.G., Rahmanian K.P., Ledford C.J.W., Carek P.J. (2018). Professional Identity, Job Satisfaction, and Commitment of Nonphysician Faculty in Academic Family Medicine. Fam. Med..

[58] Molero Jurado M.D.M., Pérez-Fuentes M.D.C., Oropesa Ruiz N.F., Simón Márquez M.D.M., Gázquez Linares J.J. (2019). Self-Efficacy and Emotional Intelligence as Predictors of Perceived Stress in Nursing Professionals. Medicina.

[59] Barnett M.J., Lindfelt T., Doroudgar S., Chan E., Ip E.J. (2022). Pharmacy-faculty work-life balance and career satisfaction: Comparison of national survey results from 2012 and 2018. Explor. Res. Clin. Soc. Pharm..

[60] Adrian C.M., Cox S.S., Phelps L.D., Schuldt B.A., Totten J.W. (2014). Issues Causing Stress among Business Faculty Members. J. Acad. Adm. High. Educ..

[61] Kennedy D.R., Clapp P., DeLuca J.L., Filtz T.M., Kroon L., Lamberts J.T., Oliphant C.M., Prescott W.A., Ray S.D. (2022). Enhancing Pharmacy Faculty Well-Being and Productivity While Reducing Burnout. Am. J. Pharm. Educ..

[62] Darbishire P., Isaacs A.N., Miller M.L. (2020). Faculty Burnout in Pharmacy Education. Am. J. Pharm. Educ..

[63] Lashuel H.A. (2020). The busy lives of academics have hidden costs—and universities must take better care of their faculty members. Nature.

[64] Price M., Salzer M.S., O’Shea A., Kerschbaum S.L. (2017). Disclosure of mental disability by college and university faculty: The negotiation of accommodations, supports, and barriers. Disabil. Stud. Q..

[65] Jayman M., Glazzard J., Rose A. Tipping point: The staff wellbeing crisis in higher education. Proceedings of the Frontiers in Education.

[66] Lashuel H. (2019). Times Higher Education. The Mental Health Crisis Must Be Met with an Accepting Campus Community. https://www.timeshighereducation.com/blog/mental-health-crisis-must-be-met-accepting-campus-community.

[67] Singh C., Cross W., Munro I., Jackson D. (2020). Occupational stress facing nurse academics-A mixed-methods systematic review. J. Clin. Nurs..

[68] Bolton D., Bhugra D. (2021). Changes in society and young people’s mental health(1). Int. Rev. Psychiatry.

[69] Zamora C., Huisman T., Ho M.L. (2022). Academic Onboarding: A Practical Guide for the Junior, Early Career Radiologist. Acad. Radiol..

[70] Mamiseishvili K., Miller M.T., Lee D. (2016). Beyond teaching and research: Faculty perceptions of service roles at research universities. Innov. High. Educ..

[71] Gold K.J. (2017). The Allure of Tenure. Acad. Med..

[72] Teshima J., McKean A.J.S., Myint M.T., Aminololama-Shakeri S., Joshi S.V., Seritan A.L., Hilty D.M. (2019). Developmental Approaches to Faculty Development. Psychiatr. Clin. North Am..

[73] Walzer S. (2010). Crossing generational divides: Experiences of new faculty in higher education. Metrop. Univ..

[74] Denson N., Szelényi K. (2022). Faculty perceptions of work-life balance: The role of marital/relationship and family status. High. Educ..

[75] Azevedo L., Shi W., Medina P.S., Bagwell M.T. (2020). Examining junior faculty work-life balance in public affairs programs in the United States. J. Public Aff. Educ..

[76] Gushulak C.A., Bodmer P., Carvalho C.R., Gladstone-Gallagher R.V., Lee Y.P., Lowman H.E., Oguguah N.M., Meinikmann K., Rii Y.M., Rodríguez-Cardona B.M. (2023). The Silent Mental Health and Well-Being Crisis of Early Career Researchers in Aquatic Sciences. Limnology.

[77] Miller A.N., Taylor S.G., Bedeian A.G. (2011). Publish or perish: Academic life as management faculty live it. Career Dev. Int..

[78] Woolston C. (2020). Postdoc survey reveals disenchantment with working life. Nature.

[79] Welch A.G., Bolin J., Reardon D. (2019). Mid-Career Faculty: Trends, Barriers, and Possibilities.

[80] DeFelippo A.M., Giles D.E. (2016). Mid-career faculty and high levels of community engagement: Intentional reshaping of meaningful careers. Int. J. Res. Serv. -Learn. Community Engagem..

[81] Grant-Vallone E.J., Ensher E.A. (2017). Re-Crafting Careers for Mid-Career Faculty: A Qualitative Study. J. High. Educ. Theory Pract..

[82] Campion M.W., Bhasin R.M., Beaudette D.J., Shann M.H., Benjamin E.J. (2016). Mid-career faculty development in academic medicine: How does it impact faculty and institutional vitality?. J. Fac. Dev..

[83] Canale A.M., Herdklotz C., Wild L. (2013). Mid-career faculty support: The middle years of the academic profession. Fac. Career Dev. Serv. Wallace Cent. Rochester Inst. Technol..

[84] Onyishi I.E., Ugwu F.O., Onyishi C.N., Okwueze F.O. (2018). Job demands and psychological well-being: Moderating role of occupational self-efficacy and job social support among mid-career academics. J. Psychol. Afr..

[85] Baker-Fletcher K., Carr D., Menn E., Ramsay N.J. (2005). Taking stock at mid-career: Challenges and opportunities for faculty. Teach. Theol. Relig..

[86] Herbert D.L., Coveney J., Clarke P., Graves N., Barnett A.G. (2014). The impact of funding deadlines on personal workloads, stress and family relationships: A qualitative study of Australian researchers. BMJ Open.

[87] Piano M., Diemer K., Hall M., Hui F., Kefalianos E., Lawford B.J., McKibbin G., Jarden R.J. (2023). A rapid review of challenges and opportunities related to diversity and inclusion as experienced by early and mid-career academics in the medicine, dentistry and health sciences fields. BMC Med. Educ..

[88] Russell B.C. (2010). Stress in Senior Faculty Careers. New Dir. High. Educ..

[89] Zeig M.J., Baldwin R.G. (2013). 5: Keeping the fire burning: Strategies to support senior faculty. Improv. Acad..

[90] Shore W.B., Gjerde C., Stearns J.A., Frey J.J. (2010). Mentoring and career transition needs of senior faculty in family medicine. Fam. Med..

[91] Stearns J., Everard K.M., Gjerde C.L., Stearns M., Shore W. (2013). Understanding the needs and concerns of senior faculty in academic medicine: Building strategies to maintain this critical resource. Acad. Med..

[92] Xu Y., Wang Y. (2023). Job stress and university faculty members’ life satisfaction: The mediating role of emotional burnout. Front. Psychol..

[93] Lee J., Kim E., Wachholtz A. (2016). The effect of perceived stress on life satisfaction: The mediating effect of self-efficacy. Chongsonyonhak Yongu.

[94] Sabagh Z., Saroyan A. (2014). Professors’ perceived barriers and incentives for teaching improvement. Int. Educ. Res..

[95] Brownell S.E., Tanner K.D. (2012). Barriers to faculty pedagogical change: Lack of training, time, incentives, and... tensions with professional identity?. CBE Life Sci. Educ..

[96] McDonald G. (2013). Does size matter? The impact of student–staff ratios. J. High. Educ. Policy Manag..

[97] Vértiz-Osores J.J., Vílchez Ochoa G.L., Vértiz-Osores R.I., Damián-Núñez E., Chico Tasayco H., Rodríguez-Fuentes A. (2019). Teacher Discomfort: Reflections on the Low Academic Performance of University Students. J. Educ. Psychol. -Propos. Y Represent..

[98] de Ruiter J.A., Poorthuis A.M., Aldrup K., Koomen H.M. (2020). Teachers’ emotional experiences in response to daily events with individual students varying in perceived past disruptive behavior. J. Sch. Psychol..

[99] Aldrup K., Klusmann U., Lüdtke O., Göllner R., Trautwein U. (2018). Student misbehavior and teacher well-being: Testing the mediating role of the teacher-student relationship. Learn. Instr..

[100] Spilt J.L., Koomen H.M., Thijs J.T. (2011). Teacher wellbeing: The importance of teacher–student relationships. Educ. Psychol. Rev..

[101] Colon-Gonzalez M.C., El Rayess F., Guevara S., Anandarajah G. (2015). Successes, challenges and needs regarding rural health medical education in continental Central America: A literature review and narrative synthesis. Rural. Remote. Health.

[102] LeBlanc C., Sonnenberg L.K., King S., Busari J. (2020). Medical education leadership: From diversity to inclusivity. GMS J. Med. Educ..

[103] Seldin P., Miller J.E., Seldin C.A. (2010). The Teaching Portfolio: A Practical Guide to Improved Performance and Promotion/Tenure Decisions.

[104] Hornstein H.A. (2017). Student evaluations of teaching are an inadequate assessment tool for evaluating faculty performance. Cogent Educ..

[105] Lakeman R., Coutts R., Hutchinson M., Lee M., Massey D., Nasrawi D., Fielden J. (2022). Appearance, insults, allegations, blame and threats: An analysis of anonymous non-constructive student evaluation of teaching in Australia. Assess. Eval. High. Educ..

[106] Heffernan T., Bosetti L. (2021). Incivility: The new type of bullying in higher education. Camb. J. Educ..

[107] Lakeman R., Coutts R., Hutchinson M., Massey D., Nasrawi D., Fielden J., Lee M. (2022). Stress, distress, disorder and coping: The impact of anonymous student evaluation of teaching on the health of higher education teachers. Assess. Eval. High. Educ..

[108] Jones E., Priestley M., Brewster L., Wilbraham S.J., Hughes G., Spanner L. (2021). Student wellbeing and assessment in higher education: The balancing act. Assess. Eval. High. Educ..

[109] Harding S., Morris R., Gunnell D., Ford T., Hollingworth W., Tilling K., Evans R., Bell S., Grey J., Brockman R. (2019). Is teachers’ mental health and wellbeing associated with students’ mental health and wellbeing?. J. Affect. Disord..

[110] Kiltz L., Rinas R., Daumiller M., Fokkens-Bruinsma M., Jansen E.P. (2020). ‘When They Struggle, I Cannot Sleep Well Either’: Perceptions and Interactions Surrounding University Student and Teacher Well-Being. Front. Psychol..

[111] Huang C., Wang Y., Li X., Ren L., Zhao J., Hu Y., Zhang L., Fan G., Xu J., Gu X. (2020). Clinical features of patients infected with 2019 novel coronavirus in Wuhan, China. Lancet.

[112] World Health Organization (2020). Director-General’s Opening Remarks at the Media Briefing on COVID-19. https://www.who.int/dg/speeches/detail/who-director-general-s-opening-remarks-at-the-media-briefing-on-covid-19---11-march-2020.

[113] United Nations (2023). WHO Chief Declares End to COVID-19 as a Global Health Emergency. https://news.un.org/en/story/2023/05/1136367.

[114] MacIntyre C.R. (2020). On a knife’s edge of a COVID-19 pandemic: Is containment still possible?. Public Health Res. Pr..

[115] Barbuddhe S.B., Rawool D.B., Gaonkar P.P., Vergis J., Dhama K., Malik S.S. (2020). Global scenario, public health concerns and mitigation strategies to counter current ongoing SARS-CoV-2 / COVID-19 pandemic. Hum Vaccin. Immunother..

[116] Shi L., Lu Z.A., Que J.Y., Huang X.L., Liu L., Ran M.S., Gong Y.M., Yuan K., Yan W., Sun Y.K. (2020). Prevalence of and Risk Factors Associated With Mental Health Symptoms Among the General Population in China During the Coronavirus Disease 2019 Pandemic. JAMA Netw. Open.

[117] Younes S., Safwan J., Rahal M., Hammoudi D., Akiki Z., Akel M. (2022). Effect of COVID-19 on mental health among the young population in Lebanon. Encephale.

[118] Lindert J., Jakubauskiene M., Bilsen J. (2021). The COVID-19 disaster and mental health-assessing, responding and recovering. Eur. J. Public. Health.

[119] Naciri A., Radid M., Kharbach A., Chemsi G. (2021). E-learning in health professions education during the COVID-19 pandemic: A systematic review. J. Educ. Eval. Health Prof..

[120] Paravattil B., Zolezzi M., Carr A.S., Al-Moslih A. (2021). Reshaping experiential education within Qatar University’s Health Programs during the COVID-19 pandemic. Qatar. Med. J..

[121] Hammoudi Halat D., Younes S., Safwan J., Akiki Z., Akel M., Rahal M. (2022). Pharmacy Students’ Mental Health and Resilience in COVID-19: An Assessment after One Year of Online Education. Eur. J. Investig. Health Psychol. Educ..

[122] Hammoudi Halat D., Safwan J., Akel M., Rahal M. (2022). Programme Description: Pharmacy education shift during times of pandemic and collapse: A perspective from a school of pharmacy in Lebanon. Pharm. Educ..

[123] Purcell W.M., Lumbreras J. (2021). Higher education and the COVID-19 pandemic: Navigating disruption using the sustainable development goals. Discov. Sustain..

[124] Rabbani M.A., Shahab Syed H.B., Ikram F. (2022). Comparison of Assessment Tools in Online and On-Campus Undergraduate Medical Examinations amidst COVID-19 Pandemic. J. Coll. Physicians Surg. Pak..

[125] Brazeau G.A. (2020). Lessons Learned and Brighter Opportunities for Pharmacy Education Amid COVID-19. Am. J. Pharm. Educ..

[126] Brazeau G.A., Frenzel J.E., Prescott W.A. (2020). Facilitating Wellbeing in a Turbulent Time. Am. J. Pharm. Educ..

[127] Carr E., Davis K., Bergin-Cartwright G., Lavelle G., Leightley D., Oetzmann C., Polling C., Stevelink S.A.M., Wickersham A., Razavi R. (2022). Mental health among UK university staff and postgraduate students in the early stages of the COVID-19 pandemic. Occup. Env. Med..

[128] Goldstein E., Topitzes J., Brown R.L., Jussel A.B. (2023). Mental health among university employees during the COVID-19 pandemic: The role of previous life trauma and current posttraumatic stress symptoms. Psychol. Trauma..

[129] Akour A., Al-Tammemi A.B., Barakat M., Kanj R., Fakhouri H.N., Malkawi A., Musleh G. (2020). The Impact of the COVID-19 Pandemic and Emergency Distance Teaching on the Psychological Status of University Teachers: A Cross-Sectional Study in Jordan. Am. J. Trop. Med. Hyg..

[130] Ma K., Liang L., Chutiyami M., Nicoll S., Khaerudin T., Ha X.V. (2022). COVID-19 pandemic-related anxiety, stress, and depression among teachers: A systematic review and meta-analysis. Work.

[131] Carr E., Oetzmann C., Davis K., Bergin-Cartwright G., Dorrington S., Lavelle G., Leightley D., Polling C., Stevelink S.A.M., Wickersham A. (2022). Trajectories of mental health among UK university staff and postgraduate students during the pandemic. Occup. Env. Med..

[132] Ozamiz-Etxebarria N., Idoiaga Mondragon N., Bueno-Notivol J., Pérez-Moreno M., Santabárbara J. (2021). Prevalence of Anxiety, Depression, and Stress among Teachers during the COVID-19 Pandemic: A Rapid Systematic Review with Meta-Analysis. Brain Sci..

[133] Hammoudi Halat D., Safwan J., Rahal M. (2023). Gendered Experiences in Pharmacy Education Exposed Through a National Financial Collapse–A Reflection From the Lebanese Crisis. Am. J. Pharm. Educ..

[134] Zheng M., Asif M., Tufail M.S., Naseer S., Khokhar S.G., Chen X., Naveed R.T. (2022). Covid academic pandemic: Techno stress faced by teaching staff for online academic activities. Front. Psychol..

[135] Salo M., Pirkkalainen H., Koskelainen T. (2019). Technostress and social networking services: Explaining users’ concentration, sleep, identity, and social relation problems. Inf. Syst. J..

[136] Li L., Wang X. (2021). Technostress inhibitors and creators and their impacts on university teachers’ work performance in higher education. Cogn. Technol. Work.

[137] Coleman M.E. (2022). Mental Health in the College Classroom: Best Practices for Instructors. Teach. Sociol..

[138] Wojtera B., Singh N., Iankovitch S., Post L., Ahmed A.A., Abouzid M. (2022). Changes in psychological distress among Polish medical university teachers during the COVID-19 pandemic. PLoS ONE.

[139] Almhdawi K.A., Obeidat D., Kanaan S.F., Hajela N., Bsoul M., Arabiat A., Alazrai A., Jaber H., Alrabbaie H. (2021). University professors’ mental and physical well-being during the COVID-19 pandemic and distance teaching. Work.

[140] Vlachopoulos D. (2022). How the “lessons learned” from emergency remote teaching can enrich European higher education in the post-COVID-19 era. High. Learn. Res. Commun..

[141] Javakhishvili J.D., Arnberg F., Greenberg N., Kazlauskas E., Lotzin A., Xavier M. (2022). Dealing with the COVID-19 pandemic in Europe: Five lessons from the European Society for Traumatic Stress Studies. Eur. J. Psychotraumatology.

[142] Levin J.S., Aliyeva A., Walker L. (2016). From community college to university: Institutionalization and neoliberalism in British Columbia and Alberta. Can. J. High. Educ..

[143] Welch P. (2021). Mass Higher Education in England—A Success Story?. Postdigital Sci. Educ..

[144] Guilbault M. (2016). Students as customers in higher education: Reframing the debate. J. Mark. High. Educ..

[145] Naylor R., Dollinger M., Mahat M., Khawaja M. (2021). Students as customers versus as active agents: Conceptualising the student role in governance and quality assurance. High. Educ. Res. Dev..

[146] Singleton-Jackson J.A., Jackson D.L., Reinhardt J. (2011). Academic Entitlement: Exploring Definitions and Dimensions of Entitled Students. Int. J. Interdiscip. Soc. Sci..

[147] Cain J., Romanelli F., Smith K.M. (2012). Academic entitlement in pharmacy education. Am. J. Pharm. Educ..

[148] Knepp K.A., Knepp M.M. (2022). Academic entitlement decreases engagement in and out of the classroom and increases classroom incivility attitudes. Soc. Psychol. Educ..

[149] Jiang L., Tripp T.M., Hong P.Y. (2017). College instruction is not so stress free after all: A qualitative and quantitative study of academic entitlement, uncivil behaviors, and instructor strain and burnout. Stress Health.

[150] Heffernan K., Gates T.G. (2018). Perceptions of teaching staff in human services about academic entitlement: Implications for staff well-being, education, and research. J. Appl. Res. High. Educ..

[151] Doyran F., Hacıfazlıoğlu Ö. (2021). In Between Wellness and Excessive Entitlement: Voices of Faculty Members. Understanding Excessive Teacher and Faculty Entitlement.

[152] Whitley R., Gläser J. (2014). The impact of institutional reforms on the nature of universities as organisations. Organizational Transformation and Scientific Change: The Impact of Institutional Restructuring on Universities and Intellectual Innovation.

[153] Howells J.R., Karataş-Özkan M., Yavuz Ç., Atiq M. (2014). University management and organisational change: A dynamic institutional perspective. Camb. J. Reg. Econ. Soc..

[154] Holloway K., Herder M. (2019). A responsibility to commercialize? Tracing academic researchers’ evolving engagement with the commercialization of biomedical research. J. Responsible Innov..

[155] Mercelis J., Galvez-Behar G., Guagnini A. (2017). Commercializing science: Nineteenth-and twentieth-century academic scientists as consultants, patentees, and entrepreneurs. Hist. Technol..

[156] Lewin T. (2013). As interest fades in the humanities, colleges worry. The New York Times.

[157] Huang J., Nigatu Y.T., Smail-Crevier R., Zhang X., Wang J. (2018). Interventions for common mental health problems among university and college students: A systematic review and meta-analysis of randomized controlled trials. J. Psychiatr. Res..

[158] Liu X.Q., Guo Y.X., Xu Y. (2023). Risk factors and digital interventions for anxiety disorders in college students: Stakeholder perspectives. World J Clin Cases.

[159] Li Y., Wang A., Wu Y., Han N., Huang H. (2021). Impact of the COVID-19 Pandemic on the Mental Health of College Students: A Systematic Review and Meta-Analysis. Front. Psychol..

[160] González-Valero G., Zurita-Ortega F., Ubago-Jiménez J.L., Puertas-Molero P. (2019). Use of Meditation and Cognitive Behavioral Therapies for the Treatment of Stress, Depression and Anxiety in Students. A Systematic Review and Meta-Analysis. Int. J. Environ. Res. Public Health.

[161] Barnett P., Arundell L.L., Saunders R., Matthews H., Pilling S. (2021). The efficacy of psychological interventions for the prevention and treatment of mental health disorders in university students: A systematic review and meta-analysis. J. Affect. Disord..

[162] Dutta A., Sharma A., Torres-Castro R., Pachori H., Mishra S. (2021). Mental health outcomes among health-care workers dealing with COVID-19/severe acute respiratory syndrome coronavirus 2 pandemic: A systematic review and meta-analysis. Indian J. Psychiatry.

[163] Ghahramani S., Kasraei H., Hayati R., Tabrizi R., Marzaleh M.A. (2022). Health care workers’ mental health in the face of COVID-19: A systematic review and meta-analysis. Int. J. Psychiatry Clin. Pract..

[164] Labrague L.J. (2021). Pandemic fatigue and clinical nurses’ mental health, sleep quality and job contentment during the COVID-19 pandemic: The mediating role of resilience. J. Nurs. Manag..

[165] Safwan J., Halat D.H., Akel M., Younes S., Rahal M., Mourad N., Akiki Z., Cherfane M., Saade F., Bouraad E. (2023). The impact of COVID-19 on the mental health of Lebanese pharmacists: A national cross-sectional study. Front. Public. Health.

[166] Manaf M.R.A., Shaharuddin M.A., Nawi A.M., Tauhid N.M., Othman H., Rahman M.R.A., Yusoff H.M., Safian N., Ng P.Y., Manaf Z.A. (2021). Perceived Symptoms of Depression, Anxiety and Stress amongst Staff in a Malaysian Public University: A Workers Survey. Int. J. Environ. Res. Public Health.

[167] Ganji K.K., Alam M.K., Siddiqui A.A., Munisekhar M.S., Alduraywish A. (2022). COVID-19 and stress: An evaluation using Beck’s depression and anxiety inventory among college students and faculty members of Jouf University. Work.

[168] Melnyk B.M., Strait L.A., Beckett C., Hsieh A.P., Messinger J., Masciola R. (2023). The state of mental health, burnout, mattering and perceived wellness culture in Doctorally prepared nursing faculty with implications for action. Worldviews Evid. Based Nurs..

[169] Costa A.J., Labuda Schrop S., McCord G., Ritter C. (2005). Depression in family medicine faculty. Fam. Med..

[170] Zambrana R.E., Valdez R.B., Pittman C.T., Bartko T., Weber L., Parra-Medina D. (2021). Workplace stress and discrimination effects on the physical and depressive symptoms of underrepresented minority faculty. Stress Health.

[171] Lal A., Tharyan A., Tharyan P. (2020). The prevalence, determinants and the role of empathy and religious or spiritual beliefs on job stress, job satisfaction, coping, burnout, and mental health in medical and surgical faculty of a teaching hospital: A cross-sectional survey. Rev. Med. Interne.

[172] Ip E.J., Lindfelt T.A., Tran A.L., Do A.P., Barnett M.J. (2020). Differences in Career Satisfaction, Work-life Balance, and Stress by Gender in a National Survey of Pharmacy Faculty. J. Pharm. Pract..

[173] Lindfelt T.A., Ip E.J., Barnett M.J. (2015). Survey of career satisfaction, lifestyle, and stress levels among pharmacy school faculty. Am. J. Health Syst. Pharm..

[174] Kabito G.G., Wami S.D., Chercos D.H., Mekonnen T.H. (2020). Work-related Stress and Associated Factors among Academic Staffs at the University of Gondar, Northwest Ethiopia: An Institution-based Cross-sectional Study. Ethiop. J. Health Sci..

[175] Seo J.H., Bae H.O., Kim B.J., Huh S., Ahn Y.J., Jung S.S., Kim C., Im S., Kim J.B., Cho S.J. (2022). Burnout of Faculty Members of Medical Schools in Korea. J. Korean Med. Sci..

[176] Ganeshan D., Wei W., Yang W. (2019). Burnout in Chairs of Academic Radiology Departments in the United States. Acad. Radiol..

[177] El-Ibiary S.Y., Yam L., Lee K.C. (2017). Assessment of Burnout and Associated Risk Factors Among Pharmacy Practice Faculty in the United States. Am. J. Pharm. Educ..

[178] (2021). Global prevalence and burden of depressive and anxiety disorders in 204 countries and territories in 2020 due to the COVID-19 pandemic. Lancet.

[179] World Health Organization Mental Disorders. June 2022..

[180] (2017). Depression and Other Common Mental Disorders: Global Health Estimates.

[181] van der Wal J.M., van Borkulo C.D., Deserno M.K., Breedvelt J.J.F., Lees M., Lokman J.C., Borsboom D., Denys D., van Holst R.J., Smidt M.P. (2021). Advancing urban mental health research: From complexity science to actionable targets for intervention. Lancet Psychiatry.

[182] World Health Organization Depressive Disorder (Depression). March 2023. https://www.who.int/news-room/fact-sheets/detail/depression.

[183] Tran T.D., Tran T., Fisher J. (2013). Validation of the depression anxiety stress scales (DASS) 21 as a screening instrument for depression and anxiety in a rural community-based cohort of northern Vietnamese women. BMC Psychiatry.

[184] Makara-Studzińska M., Tyburski E., Załuski M., Adamczyk K., Mesterhazy J., Mesterhazy A. (2021). Confirmatory Factor Analysis of Three Versions of the Depression Anxiety Stress Scale (DASS-42, DASS-21, and DASS-12) in Polish Adults. Front Psychiatry.

[185] Ali A.M., Alkhamees A.A., Hori H., Kim Y., Kunugi H. (2021). The Depression Anxiety Stress Scale 21: Development and Validation of the Depression Anxiety Stress Scale 8-Item in Psychiatric Patients and the General Public for Easier Mental Health Measurement in a Post COVID-19 World. Int. J. Environ. Res. Public Health.

[186] Ali A.M., Hori H., Kim Y., Kunugi H. (2022). The Depression Anxiety Stress Scale 8-Items Expresses Robust Psychometric Properties as an Ideal Shorter Version of the Depression Anxiety Stress Scale 21 Among Healthy Respondents From Three Continents. Front. Psychol..

[187] Beck A.T., Epstein N., Brown G., Steer R.A. (1988). An inventory for measuring clinical anxiety: Psychometric properties. J. Consult. Clin. Psychol..

[188] Quinn B.L., Peters A. (2017). Strategies to Reduce Nursing Student Test Anxiety: A Literature Review. J. Nurs. Educ..

[189] Aloufi M.A., Jarden R.J., Gerdtz M.F., Kapp S. (2021). Reducing stress, anxiety and depression in undergraduate nursing students: Systematic review. Nurse Educ. Today.

[190] Domínguez-Solís E., Lima-Serrano M., Lima-Rodríguez J.S. (2021). Non-pharmacological interventions to reduce anxiety in pregnancy, labour and postpartum: A systematic review. Midwifery.

[191] Salari N., Khazaie H., Hosseinian-Far A., Khaledi-Paveh B., Kazeminia M., Mohammadi M., Shohaimi S., Daneshkhah A., Eskandari S. (2020). The prevalence of stress, anxiety and depression within front-line healthcare workers caring for COVID-19 patients: A systematic review and meta-regression. Hum. Resour. Health.

[192] World Health Organization (2023). Stress. https://www.who.int/news-room/questions-and-answers/item/stress.

[193] Zhang M., Murphy B., Cabanilla A., Yidi C. (2021). Physical relaxation for occupational stress in healthcare workers: A systematic review and network meta-analysis of randomized controlled trials. J. Occup. Health.

[194] Kivimäki M., Virtanen M., Elovainio M., Kouvonen A., Väänänen A., Vahtera J. (2006). Work stress in the etiology of coronary heart disease--a meta-analysis. Scand. J. Work Environ. Health.

[195] Gmelch W.H., Wilke P.K., Lovrich N.P. (1986). Dimensions of stress among university faculty: Factor-analytic results from a national study. Res. High. Educ..

[196] Iqbal A., Kokash H. (2011). Faculty Perception of Stress and Coping Strategies in a Saudi Private University: An Exploratory Study. Int. Educ. Stud..

[197] Ashley G., Cort M. (2007). The effects of the practice of the Newstart Health Regimen on faculty stress among faculty at Seventh-Day Adventist colleges and universities. Christ. High. Educ..

[198] Pr G., Asokan S., Viswanath S. (2021). Job satisfaction and stress among dental faculty members: A mixed-method approach. J. Dent. Educ..

[199] Shahrour G., Dardas L.A. (2020). Acute stress disorder, coping self-efficacy and subsequent psychological distress among nurses amid COVID-19. J. Nurs. Manag..

[200] Bohlken J., Schömig F., Lemke M.R., Pumberger M., Riedel-Heller S.G. (2020). COVID-19 Pandemic: Stress Experience of Healthcare Workers—A Short Current Review. Psychiatr. Prax..

[201] Garcia R., Paraiso L.O., Sy-Luna G., Laraño L. (2021). Impact of covid19 pandemic on work-related stress among university faculty: A longitudinal study. Int. J. Recent Adv. Multidiscipl. Res.

[202] Shrivastava A., Shukla N. (2017). A critical review on occupational stress factors affecting faculty members working in higher educational institutions in India. Pac. Bus. Rev. Int..

[203] Slišković A., Maslić Seršić D. (2011). Work stress among university teachers: Gender and position differences. Arh. Hig. Rada Toksikol..

[204] Freudenberger H.J. (1974). Staff Burn-Out. J. Soc. Issues.

[205] Nunn K., Isaacs D. (2019). Burnout. J. Paediatr. Child Health.

[206] Epstein E.G., Haizlip J., Liaschenko J., Zhao D., Bennett R., Marshall M.F. (2020). Moral distress, mattering, and secondary traumatic stress in provider burnout: A call for moral community. AACN Adv. Crit. Care.

[207] Alrawashdeh H.M., Al-Tammemi A.B., Alzawahreh M.K., Al-Tamimi A., Elkholy M., Al Sarireh F., Abusamak M., Elehamer N.M.K., Malkawi A., Al-Dolat W. (2021). Occupational burnout and job satisfaction among physicians in times of COVID-19 crisis: A convergent parallel mixed-method study. BMC Public Health.

[208] Sales P.M.G., Arshed A., Cosmo C., Li P., Garrett M., Cohen M.A. (2021). Burnout and Moral Injury Among Consultation-Liaison Psychiatry Trainees. Psychodyn. Psychiatry.

[209] Gómez-Polo C., Casado A.M.M., Montero J. (2022). Burnout syndrome in dentists: Work-related factors. J. Dent..

[210] Kariou A., Koutsimani P., Montgomery A., Lainidi O. (2021). Emotional Labor and Burnout among Teachers: A Systematic Review. Int. J. Environ. Res. Public Health.

[211] Edú-Valsania S., Laguía A., Moriano J.A. (2022). Burnout: A Review of Theory and Measurement. Int. J. Environ. Res. Public Health.

[212] Maslach burnout inventory (MBI) Mind Garden. http://www.mindgarden.com/products/mbi.htm.

[213] Maslach C., Schaufeli W.B., Leiter M.P. (2001). Job burnout. Annu. Rev. Psychol..

[214] Obregon M., Luo J., Shelton J., Blevins T., MacDowell M. (2020). Assessment of burnout in medical students using the Maslach Burnout Inventory-Student Survey: A cross-sectional data analysis. BMC Med. Educ..

[215] Hosseini M., Soltanian M., Torabizadeh C., Shirazi Z.H. (2022). Prevalence of burnout and related factors in nursing faculty members: A systematic review. J. Educ. Eval. Health Prof..

[216] Kichloo A. (2020). Early Mentoring for Research and Academic Careers. J. Investig. Med. High Impact Case Rep..

[217] Melnyk B.M., Hsieh A.P., Tan A., Gawlik K.S., Hacker E.D., Ferrell D., Simpson V., Burda C., Hagerty B., Scott L.D. (2021). The state of mental health and healthy lifestyle behaviors in nursing, medicine and health sciences faculty and students at Big 10 Universities with implications for action. J. Prof. Nurs..

[218] Parola V., Coelho A., Cardoso D., Sandgren A., Apóstolo J. (2017). Prevalence of burnout in health professionals working in palliative care: A systematic review. JBI Database Syst. Rev. Implement Rep..

[219] Elbarazi I., Loney T., Yousef S., Elias A. (2017). Prevalence of and factors associated with burnout among health care professionals in Arab countries: A systematic review. BMC Health Serv. Res..

[220] Xiao Y., Wang J., Chen S., Wu Z., Cai J., Weng Z., Li C., Zhang X. (2014). Psychological distress, burnout level and job satisfaction in emergency medicine: A cross-sectional study of physicians in China. Emerg. Med. Australas..

[221] Lee K.C., Ye G.Y., Choflet A., Barnes A., Zisook S., Ayers C., Davidson J.E. (2022). Longitudinal analysis of suicides among pharmacists during 2003-2018. J. Am. Pharm. Assoc..

[222] Stelnicki A.M., Carleton R.N. (2021). Nursing Leadership Has an Important Role in the Management of Nurses’ Mental Health. Nurs. Lead..

[223] Hussain R., Wark S., Dillon G., Ryan P. (2016). Self-reported physical and mental health of Australian carers: A cross-sectional study. BMJ Open.

[224] Zhou Y., Wang W., Sun Y., Qian W., Liu Z., Wang R., Qi L., Yang J., Song X., Zhou X. (2020). The prevalence and risk factors of psychological disturbances of frontline medical staff in china under the COVID-19 epidemic: Workload should be concerned. J. Affect. Disord..

[225] Hummel S., Oetjen N., Du J., Posenato E., Resende de Almeida R.M., Losada R., Ribeiro O., Frisardi V., Hopper L., Rashid A. (2021). Mental Health Among Medical Professionals During the COVID-19 Pandemic in Eight European Countries: Cross-sectional Survey Study. J. Med. Internet Res..

[226] Raudenská J., Steinerová V., Javůrková A., Urits I., Kaye A.D., Viswanath O., Varrassi G. (2020). Occupational burnout syndrome and post-traumatic stress among healthcare professionals during the novel coronavirus disease 2019 (COVID-19) pandemic. Best Pract. Res. Clin. Anaesthesiol..

[227] Della Monica A., Ferrara P., Dal Mas F., Cobianchi L., Scannapieco F., Ruta F. (2022). The impact of Covid-19 healthcare emergency on the psychological well-being of health professionals: A review of literature. Ann. Ig..

[228] Lee P., Miller M.T., Kippenbrock T.A., Rosen C., Emory J. (2017). College nursing faculty job satisfaction and retention: A national perspective. J. Prof. Nurs..

[229] Wallace J.E., Lemaire J.B., Ghali W.A. (2009). Physician wellness: A missing quality indicator. Lancet.

[230] Melnyk B.M., Tan A., Hsieh A.P., Gawlik K., Arslanian-Engoren C., Braun L.T., Dunbar S., Dunbar-Jacob J., Lewis L.M., Millan A. (2021). Critical Care Nurses’ Physical and Mental Health, Worksite Wellness Support, and Medical Errors. Am. J. Crit. Care.

[231] Schattner A., Rudin D., Jellin N. (2004). Good physicians from the perspective of their patients. BMC Health Serv. Res..

[232] Moss P.J., Lambert T.W., Goldacre M.J., Lee P. (2004). Reasons for considering leaving UK medicine: Questionnaire study of junior doctors’ comments. BMJ.

[233] Chichra A., Abhijnhan A., Tharyan P. (2019). Job stress and satisfaction in faculty of a teaching hospital in south India: A cross-sectional survey. J. Postgrad. Med..

[234] Gabbe S.G., Hagan Vetter M., Nguyen M.C., Moffatt-Bruce S., Fowler J.M. (2018). Changes in the burnout profile of chairs of academic departments of obstetrics and gynecology over the past 15 years. Am. J. Obstet. Gynecol..

[235] Fridner A., Belkić K., Marini M., Gustafsson Sendén M., Schenck-Gustafsson K. (2012). Why don’t academic physicians seek needed professional help for psychological distress?. Swiss Med. Wkly..

[236] Lindfelt T., Ip E.J., Gómez A., Barnett M.J. (2018). The impact of work-life balance on intention to stay in academia: Results from a national survey of pharmacy faculty. Res. Soc. Adm. Pharm..

[237] Boamah S.A., Kalu M., Stennett R., Belita E., Travers J. (2023). Pressures in the Ivory Tower: An Empirical Study of Burnout Scores among Nursing Faculty. Int. J. Environ. Res. Public Health.

[238] World Health Organization (2022). WHO European Framework for Action on Mental Health 2021–2025.

[239] Fernandez A., Howse E., Rubio-Valera M., Thorncraft K., Noone J., Luu X., Veness B., Leech M., Llewellyn G., Salvador-Carulla L. (2016). Setting-based interventions to promote mental health at the university: A systematic review. Int. J. Public. Health.

[240] Borg A., Sammut A., Grech A., Lautier E.C., Priebe S., Azzopardi-Muscat N. (2022). Developing the 2020-2030 mental health strategy for Malta: Addressing the needs of a small island state undergoing rapid socioeconomic transition. Health Policy.

[241] Occhipinti J.A., Buchanan J., Skinner A., Song Y.J.C., Tran K., Rosenberg S., Fels A., Doraiswamy P.M., Meier P., Prodan A. (2022). Measuring, Modeling, and Forecasting the Mental Wealth of Nations. Front. Public Health.

[242] Januário S.S., das Neves Peixoto F.S., Lima N.N., do Nascimento V.B., de Sousa D.F., Pereira Luz D.C., da Silva C.G., Rolim Neto M.L. (2017). Mental health and public policies implemented in the Northeast of Brazil: A systematic review with meta-analysis. Int. J. Soc. Psychiatry.

[243] Mapanga W., Casteleijn D., Ramiah C., Odendaal W., Metu Z., Robertson L., Goudge J. (2019). Strategies to strengthen the provision of mental health care at the primary care setting: An Evidence Map. PLoS ONE.

[244] Nordin H., Rørtveit K., Mathisen G.E., Joa I., Johannessen J.O., Ruud T., Hartveit M. (2022). Frontline leadership for implementing clinical guidelines in Norwegian mental health services: A qualitative study. J. Health Organ. Manag..

[245] Cleary M., West S., Haik J., Greenwood M., Toohey T., Kornhaber R. (2019). Capacity building in burns and mental health care. Burns.

[246] Kwak L., Toropova A., Powell B.J., Lengnick-Hall R., Jensen I., Bergström G., Elinder L.S., Stigmar K., Wåhlin C., Björklund C. (2022). A randomized controlled trial in schools aimed at exploring mechanisms of change of a multifaceted implementation strategy for promoting mental health at the workplace. Implement. Sci..

[247] Arensman E., O’Connor C., Leduc C., Griffin E., Cully G., Ní Dhálaigh D., Holland C., Van Audenhove C., Coppens E., Tsantila F. (2022). Mental Health Promotion and Intervention in Occupational Settings: Protocol for a Pilot Study of the MENTUPP Intervention. Int. J. Environ. Res. Public Health.

[248] Martin A., Karanika-Murray M., Biron C., Sanderson K. (2016). The Psychosocial Work Environment, Employee Mental Health and Organizational Interventions: Improving Research and Practice by Taking a Multilevel Approach. Stress Health.

[249] Cottini E., Lucifora C. (2013). Mental health and working conditions in Europe. ILR Rev..

[250] Tan L., Wang M.J., Modini M., Joyce S., Mykletun A., Christensen H., Harvey S.B. (2014). Preventing the development of depression at work: A systematic review and meta-analysis of universal interventions in the workplace. BMC Med..

[251] Panagioti M., Panagopoulou E., Bower P., Lewith G., Kontopantelis E., Chew-Graham C., Dawson S., van Marwijk H., Geraghty K., Esmail A. (2017). Controlled Interventions to Reduce Burnout in Physicians: A Systematic Review and Meta-analysis. JAMA Intern. Med..

[252] Deneckere S., Euwema M., Lodewijckx C., Panella M., Mutsvari T., Sermeus W., Vanhaecht K. (2013). Better interprofessional teamwork, higher level of organized care, and lower risk of burnout in acute health care teams using care pathways: A cluster randomized controlled trial. Med. Care.

[253] Bazzano A.N., Sun Y., Chavez-Gray V., Akintimehin T., Gustat J., Barrera D., Roi C. (2022). Effect of Yoga and Mindfulness Intervention on Symptoms of Anxiety and Depression in Young Adolescents Attending Middle School: A Pragmatic Community-Based Cluster Randomized Controlled Trial in a Racially Diverse Urban Setting. Int. J. Environ. Res. Public Health.

[254] Markwell P., Polivka B.J., Morris K., Ryan C., Taylor A. (2016). Snack and Relax^®^: A Strategy to Address Nurses’ Professional Quality of Life. J. Holist. Nurs..

[255] Wainberg M.L., Scorza P., Shultz J.M., Helpman L., Mootz J.J., Johnson K.A., Neria Y., Bradford J.E., Oquendo M.A., Arbuckle M.R. (2017). Challenges and Opportunities in Global Mental Health: A Research-to-Practice Perspective. Curr. Psychiatry Rep..

[256] Reavley N., Livingston J., Buchbinder R., Bennell K., Stecki C., Osborne R.H. (2010). A systematic grounded approach to the development of complex interventions: The Australian WorkHealth Program--arthritis as a case study. Soc. Sci. Med..

[257] von Thiele Schwarz U., Nielsen K., Edwards K., Hasson H., Ipsen C., Savage C., Simonsen Abildgaard J., Richter A., Lornudd C., Mazzocato P. (2021). How to design, implement and evaluate organizational interventions for maximum impact: The Sigtuna Principles. Eur. J. Work Organ. Psychol..

[258] (2019). Mind. Mentally Health Universities Programme. https://www.mind.org.uk/workplace/mentally-healthy-universities-programme/.

[259] Stahel P.F., Ahankoob N., Nguyen C. (2022). Servant leadership: An endangered species?. Patient Saf. Surg..

[260] Desselle S.P., Darbishire P.L., Clubbs B.H. (2020). Pharmacy Faculty Burnout: Cause for Concern that Requires Our Support and Use of Best Evidence. Innov. Pharm..

[261] Di Fabio A., Palazzeschi L., Bucci O. (2017). Gratitude in Organizations: A Contribution for Healthy Organizational Contexts. Front. Psychol..

[262] Sawyer K.B., Thoroughgood C.N., Stillwell E.E., Duffy M.K., Scott K.L., Adair E.A. (2022). Being present and thankful: A multi-study investigation of mindfulness, gratitude, and employee helping behavior. J. Appl. Psychol..

[263] Bechara Secchin L.S., da Silva Ezequiel O., Vitorino L.M., Lucchetti A.L.G., Lucchetti G. (2020). Implementation of a Longitudinal Mentorship Program for Quality of Life, Mental Health, and Motivation of Brazilian Medical Students. Acad. Psychiatry.

[264] Altonji S.J., Baños J.H., Harada C.N. (2019). Perceived Benefits of a Peer Mentoring Program for First-Year Medical Students. Teach. Learn. Med..

[265] Schrubbe K.F. (2004). Mentorship: A critical component for professional growth and academic success. J. Dent. Educ..

[266] Abdollahi M., Heshmati Nabavi F. (2023). Mentoring as an Appropriate Strategy for Medical Faculty Member Development in Higher Education: A Systematic Review. J. Adv. Med. Educ. Prof..

[267] Kibbe M.R., Pellegrini C.A., Townsend C.M., Helenowski I.B., Patti M.G. (2016). Characterization of Mentorship Programs in Departments of Surgery in the United States. JAMA Surg..

[268] Fuller K., Maniscalco-Feichtl M., Droege M. (2008). The role of the mentor in retaining junior pharmacy faculty members. Am. J. Pharm. Educ..

[269] Shiri R., Turunen J., Kausto J., Leino-Arjas P., Varje P., Väänänen A., Ervasti J. (2022). The Effect of Employee-Oriented Flexible Work on Mental Health: A Systematic Review. Healthcae.

[270] Eek F., Axmon A. (2013). Attitude and flexibility are the most important work place factors for working parents’ mental wellbeing, stress, and work engagement. Scand J. Public Health.

[271] Lee H.F., Chang Y.J. (2022). The Effects of Work Satisfaction and Work Flexibility on Burnout in Nurses. J. Nurs. Res..

[272] Barley S.R., Meyerson D.E., Grodal S. (2011). E-mail as a source and symbol of stress. Organ. Sci..

[273] Lanctot A., Duxbury L. (2021). When everything is urgent! Mail use and employee well-being. Comput. Hum. Behav. Rep..

[274] Kushlev K., Dunn E.W. (2015). Checking email less frequently reduces stress. Comput. Hum. Behav..

[275] Allen J.A., Sands S.J., Mueller S.L., Frear K.A., Mudd M., Rogelberg S.G. (2012). Employees’ feelings about more meetings: An overt analysis and recommendations for improving meetings. Manag. Res. Rev..

[276] LeBlanc L.A., Nosik M.R. (2019). Planning and leading effective meetings. Behav. Anal. Pract..

[277] Kennedy D.R., Porter A.L. (2022). The Illusion of Urgency. Am. J. Pharm. Educ..

[278] Dyer K.A. (2023). Daily healthy habits to reduce stress and increase longevity. J. Interprofessional Educ. Pract..

[279] (2022). WHO Guideline on Self-Care Interventions for Health and Well-Being, 2022 Revision.

[280] Savva L.I., Saigushev N.Y., Vedeneeva O.A., Pavlova L.V., Rabin E.I. (2017). Student’s time-awareness formation: Self-organized personality as promoting factor for mental health. Eur. Proc. Soc. Behav. Sci..

[281] Evans M., Fisher E.B. (2022). Social Isolation and Mental Health: The Role of Nondirective and Directive Social Support. Community Ment. Health J..

[282] Stuart J., O’Donnell K., O’Donnell A., Scott R., Barber B. (2021). Online Social Connection as a Buffer of Health Anxiety and Isolation During COVID-19. Cyberpsychol. Behav. Soc. Netw..

[283] Harandi T.F., Taghinasab M.M., Nayeri T.D. (2017). The correlation of social support with mental health: A meta-analysis. Electron. Physician.

[284] Ahmed M., Cerda I., Maloof M. (2023). Breaking the vicious cycle: The interplay between loneliness, metabolic illness, and mental health. Front. Psychiatry.

[285] Petersen A.M., Riccaboni M., Stanley H.E., Pammolli F. (2012). Persistence and uncertainty in the academic career. Proc. Natl. Acad. Sci. USA.

[286] Scior K., Rüsch N., White C., Corrigan P.W. (2020). Supporting mental health disclosure decisions: The Honest, Open, Proud programme. Br. J. Psychiatry.

[287] Thornicroft G., Mehta N., Clement S., Evans-Lacko S., Doherty M., Rose D., Koschorke M., Shidhaye R., O’Reilly C., Henderson C. (2016). Evidence for effective interventions to reduce mental-health-related stigma and discrimination. Lancet.

[288] Pemovska T., Arënliu A., Konjufca J., Uka F., Hunter J., Bajraktarov S., Stevović L.I., Jerotić S., Kulenović A.D., Novotni A. (2021). Implementing a digital mental health intervention for individuals with psychosis—A multi-country qualitative study. BMC Psychiatry.

[289] Dekker I., De Jong E.M., Schippers M.C., De Bruijn-Smolders M., Alexiou A., Giesbers B. (2020). Optimizing Students’ Mental Health and Academic Performance: AI-Enhanced Life Crafting. Front. Psychol..

